# Plant gum identification in historic artworks

**DOI:** 10.1038/srep44538

**Published:** 2017-04-20

**Authors:** Clara Granzotto, Julie Arslanoglu, Christian Rolando, Caroline Tokarski

**Affiliations:** 1 Miniaturisation pourla Synthèse, l’Analyse & la Protéomique (MSAP), USR CNRS 3290, Université de Lille 1 Sciences et Technologies, 59655 Villeneuve d’Ascq, France; 2 Department of Molecular Sciences and Nanosystems, Ca’ Foscari University, Venice, Italy; 3 Department of Scientific Research, The Metropolitan Museum of Art, New York, NY, USA

## Abstract

We describe an integrated and straightforward new analytical protocol that identifies plant gums from various sample sources including cultural heritage. Our approach is based on the identification of saccharidic fingerprints using mass spectrometry following controlled enzymatic hydrolysis. We developed an enzyme cocktail suitable for plant gums of unknown composition. Distinctive MS profiles of gums such as arabic, cherry and locust-bean gums were successfully identified. A wide range of oligosaccharidic combinations of pentose, hexose, deoxyhexose and hexuronic acid were accurately identified in gum arabic whereas cherry and locust bean gums showed respectively Pent_x_Hex_y_ and Hex_n_ profiles. Optimized for low sample quantities, the analytical protocol was successfully applied to contemporary and historic samples including ‘Colour Box Charles Roberson & Co’ dating 1870s and drawings from the American painter Arthur Dove (1880–1946). This is the first time that a gum is accurately identified in a cultural heritage sample using structural information. Furthermore, this methodology is applicable to other domains (food, cosmetic, pharmaceutical, biomedical).

The term “gum” refers to a substance containing a mixture of chemical compounds that is able to form gels that increase solution viscosity and stabilize emulsions and foams. It does not designate a class of chemical compounds^1^, however, it is commonly associated with water soluble and modified polysaccharides. Plant gums are naturally occurring from various plant cell walls, seeds, trees or shrub exudates and seaweed extracts^1^. Due to their properties, a number of plant gums such as gum arabic (from *Acacia senegal* and *seyal*) and locust bean gum (LBG) (*Ceratonia siliqua*) are widely employed in the industries of food, cosmetics, textiles and biomedical/pharmaceutics^2^,^3^,^4^,^5^. Furthermore, plant gums have also been used as binding media and adhesives in artworks and cultural artefacts^6^. Their use has been traced back to ca. 2600 B.C. through the Fourth Dynasty of Egyptian time^7^ as binders and adhesives for mineral pigments on cartonnage and in the practices of mummification. In recent centuries gums have been widely used, for example, as binders for watercolor and gouache paints.

Plant gum polysaccharides have complex structures, most of them having a high molecular mass (up to several million Dalton), highly branched structures with a large variety of monosaccharides and glycosidic linkages, high polydispersity and heterogeneity, and some have covalently attached protein moieties^8^. While polysaccharides are studied by different analytical techniques, limited information is provided in the literature about their chemical structure. The structural analyses of plant gum polysaccharides^9^ focus on the monosaccharide composition and sequences, molecular weight and distribution, linkage positions between the glycosidic linkages and branches, ring size (furanosidic/pyranosidic rings), anomeric configuration (α/β), substitutions and, identification/localization of potentially attached proteins. Considering for example gum arabic, despite its wide use in industry, its structural characterization is incomplete and subject to many corrections^10^,^11^,^12^,^13^,^14^,^15^,^16^,^17^. To elucidate its structural features, methylation-gas-chromatography mass-spectrometry (GC-MS) associated with 2D-NMR are employed. These techniques recently showed that the polysaccharide backbone of gum arabic (*Acacia senegal*) consists of 1,3-linked β-D-Gal*p* residues, substituted at O-2/O-6/O-4 positions^12^, with residues of 2,3,6-β-D-Gal*p*1, 3,4-Gal*p*1, 3,4,6-Gal*p*1 present in side chains^12^, in addition to 1,6-β-D-Gal*p* side chains to which many Ara, UA and Rha residues are linked^12^,^18^ (see saccharide nomenclature/abbreviations in Supplementary Table 1). These techniques have also highlighted the possible (partial) structure and composition of several other plant gums such as tragacanth^19^, *Acacia tortuosa*^20^ and ghatti^21^ gums. Focusing on poly/oligosaccharide analyses using MS^22^,^23^,^24^,^25^,^26^, whereas GC-MS^11^ or electrospray (ESI)-MS^10^ are commonly used for identification of, respectively, mono- and oligosaccharides following their hydrolysis (typically using Smith degradation^11^), the use of enzymes^27^,^28^,^29^,^30^,^31^ was recently evaluated to produce larger saccharidic sequences that were analyzed by ESI or matrix-assisted laser desorption/ionization (MALDI)-MS and MS/MS^28^,^29^,^30^,^31^,^32^. For example, β-mannanase was used to hydrolyze LBG^28^ and guar gum^29^ which contain β-(1–4)-Man*p* backbone. This method allowed the identification of hexoses substituted with pentose and acetylated hexoses substituted or not with pentose in the side chains of LBG backbone^28^. In another study, l-Ara oligomers (up to 10 monomers) were found to be attached to GlcA in mesquite gum^30^. An application to highly branched arabinogalactans (AGs) from *Arabidopsis thaliana* leaf, unveiled some structural features of the polysaccharide using a set of enzymes^31^ (e.g. β-(1-3)-galactan backbone with β-(1-6)-galactan side chains substituted with L-Ara*f* residues, 4-O-methyl-GlcA, L-Fuc). These mass spectrometric strategies have mainly been applied to the study of natural polymers (raw products) and they are still under development for a better understanding of the gums’ molecular structures and, therefore, their function in a specific environment (e.g. application to food/pharmaceutical industries). In addition, to our knowledge, there is currently no single and straightforward MS method to clearly identify a specific plant gum using structural features.

When moving from raw/food/pharmaceutical samples to the analysis of artworks’ materials, the identification of plant gums becomes even more challenging due to the limited amount of sample typically obtained from a museum artefact (order of tens-to-hundreds of micrograms), the possible degradation of the analyte due to the ageing and mainly the complexity of the sample (i.e. consisting of multiple organic/inorganic materials, in addition to the suspected polysaccharide). In the field of conservation science, gum identification in art materials provides important information on how the artwork was made, but it is also crucial in addressing questions of authenticity and in the development of appropriate conservation and preservation strategies. The most conventional approach employed for the discrimination of plant sources is based on the monosaccharide composition using GC-MS^33^ or pyrolysis coupled to GC-MS (Py-GC-MS)^34^ following gum hydrolysis. For example the GC-MS profile of gum arabic is characterized by a high percentage (∼40%) of both Ara and Gal (Rha ~ 10%, GlcA < 10%, no Fuc, no GalA, no Man) while karaya gum does not contain Ara. Fuc is present only in tragacanth gum so it can be used as a marker for that gum^35^. However this approach has several limitations: the procedure is time-consuming, data are often difficult to interpret and the monosaccharide profiles can be altered by the presence of other components^33^ or mixtures of different polysaccharide materials, thus hindering the correct identification of the gum/s. Enzyme-linked immunosorbent assay has also shown great potential in distinguishing gum arabic and tragacanth from proteinaceous binders in art samples^36^,^37^. More recently, combination of scanning-electron microscopy with energy-dispersive X-ray spectroscopy and X-ray photoelectron spectroscopy was used to study the surface of watercolors paints and the identification of gum arabic was suggested^38^.

In this paper a novel methodology for plant gum identification, applied to both commercially and industrially available raw gums and more complex samples from works of art, is reported. While a method, called peptide mass fingerprint, is widely known for the straightforward identification of proteins in the biomedical field^39^ and it has been successfully adapted to the study of proteins in samples from cultural heritage^40^, the identification of plant gums is still challenging and this paper aims to exceed these obstacles by adapting metabolomics-like strategies^41^. The strategy consists of the partial enzymatic digestion of the natural polymers with glycoside hydrolases followed by MALDI-TOF MS and MS/MS analysis of the released oligosaccharides. We developed an enzyme cocktail suitable for plant gums of unknown composition. This MS-based methodology informs on structural features of gums starting from very small amounts of sample. Beyond the gum structure evidence and the method’s high sensitivity, the technique is reliable and robust enabling the successful analysis of a unique microsample (generally the case considering samples from cultural heritage), even in the case of contaminations (e.g. with paper fibers). The protocol steps were developed by working on arabinogalactan and galactomannan standards and raw gum samples. The MS fingerprint profiles of gum arabic, cherry gum and LBG are reported and an attempt was made to determine the oligosaccharide composition and structure by means of combining different hydrolyzing enzymes and MALDI-TOF Collision-Induced-Dissociation (CID) experiments. Attention was particularly focused on the most commonly used gum arabic and the MS profile was confirmed by analyzing several different *Acacia senegal* variety (var.) *senegal* samples. Finally, the method was successfully applied to the investigation of commercial watercolor samples from contemporary artists’ tube-paints and, for the first time, from an historic artists’ watercolor paint set dating 1870 (Colour Box Charles Roberson & Co, The Metropolitan Museum of Art; Supplementary Fig. 1) and from two drawings by Arthur Dove, *Boat Houses* (1935) and *Study for ‘Goat’* (ca. 1934), in the collection of the Metropolitan Museum of Art.

## Results

### Protocol optimization

We propose a straightforward analytical strategy for gum identification using their mass fingerprints. The methodology is applicable to various types of samples including historic samples from cultural heritage. It is based on two main steps: the partial enzymatic hydrolysis of the polysaccharidic components and MALDI-TOF MS and MS/MS analyses for gum fingerprint identification. Initially attention was focused on the enzyme selection using two standard substrates: galactomannans and type II-AGs. Both polysaccharides are constitutive of several plant gums such as guar and LBG for galactomannans, and arabic and cherry gums for type II-AGs. Since galactomannans are characterized by a main chain of β-d-(1,4)-Man to which α-d-1,6-Gal units are attached, both α-galactosidase and endo-1,4-β-mannanase enzymes were evaluated for the hydrolysis of the polysaccharides (Table 1)^42^. As introduced previously, the type II-AGs have a more complex structure with highly branched moieties and various constitutive saccharides (β-(1,3)-Gal substituted with β-1,6-Gal*p*, Ara*f*, Ara*p*, D-GlcA, 4-O-methyl-D- GlcA and L-Rha)^18^,^43^. The complete hydrolysis of type II-AGs with enzymes has not been reported. Due to its structural complexity, several enzymes were tested for its partial hydrolysis; a first set acting on the side chains and a second set on the main chain. The sequential action of enzymes was evaluated in the following order: α-L-arabinofuranosidase, β-glucuronidase, α-L-rhamnosidase, β-galactosidase, exo-β-1,3-galactanase (Table 1). The protocol optimization includes the evaluation of various enzyme activities (from 25 to 1000 mU), digestion timing, buffer composition and pH. The hydrolysis of both standard polysaccharides was also performed without a previous removal of the side chains. For each condition the results were evaluated using MALDI-TOF MS (data not shown). Unexpectedly, experiments showed that the best results were obtained by incubating each polysaccharide with only one enzyme: exo-β-1,3-galactanase for the type II-AGs and endo-1,4-β-mannanase for the galactomannan. These results showed that a polysaccharide mass fingerprint between 600 and 2500 Da (and not an extended gum digest) could be obtained by incubating the samples with 100 mU of enzyme for 5 hours, instead of the typical 24 hours. As a further improvement of the strategy, since the prediction of the necessary enzyme/s is not feasible when dealing with a sample of unknown composition, an enzyme cocktail suitable for the digestion of all plant gums under investigation, regardless of their polysaccharide structure, was developed and used as the standard procedure. The enzyme cocktail contains exo-β-1,3-galactanase and endo-1,4-β-mannanase (Table 1) and the following results are obtained using this method. This aspect represents a methodological improvement since most of the strategies are targeted on a specific gum and there is not a common approach which allows the discrimination of gum mixtures or of unknown gum sources.

### Identification of raw plant gums mass fingerprint

#### Identification of gum mass fingerprint using MALDI-TOF MS analysis

**Figure 1:**
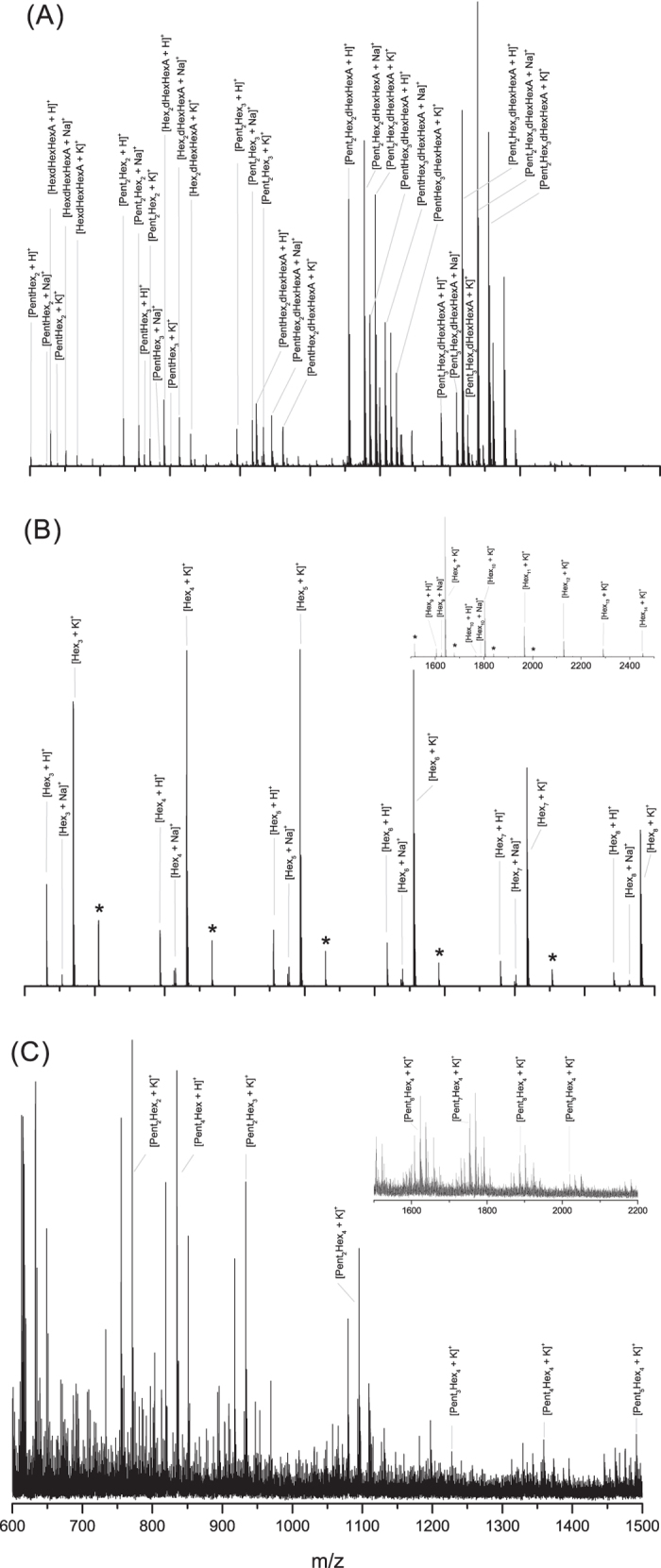
MS profiles of the enzymatically digested (**A**) gum arabic; (**B**) locust bean gum, the *m/z* range 1500–2500 Da is showed in the magnified region; and (**C**) cherry gum, the *m/z* range 1500–2200 Da is showed in the magnified region. The reported ions correspond to oligosaccharides derivatized with 3-aminoquinoline, the non-derivatized ones are indicated with an asterisk (*). The monosaccharide order for each oligosaccharide is arbitrary and does not refer to its structure. The y axis of spectra is the relative intensity.

Gum arabic from *Acacia senegal* var. *senegal,* cherry gum and LBG, which are hypothetically characterized by different structural properties (introduced previously for gum arabic^12^; galactomannans with Ara and acetyl-Hex for LBG^28^; AGs for cherry^44^), were chosen to prove the applicability of this developed method. Since these gums are not only used as artistic materials but are mainly employed in the food industry, their analysis is of considerable value. The gum samples were prepared according to the optimized protocol described in the Methods section. No desalting prior to analysis was used since the tested strategies did not show any improvement in the oligosaccharide recovery or in the signal to noise of MS spectra. The oligosaccharides were derivatized with 3-aminoquinoline (3-AQ)^45^ before MALDI-TOF analysis that was performed in positive mode. Derivatization allowed the on-target formation of Schiff bases and, despite their enhanced proton affinity, sodium and potassium adducts were also identified in the gum spectra. The MS spectra profiles of the enzymatically digested plant gums (Fig. 1) cover the 600–2500 mass range and the oligosaccharidic fingerprints consisted predominantly of hexosyl, pentosyl, deoxyhexosyl and hexuronosyl acids residues. Each gum showed a specific MS profile that allowed the discrimination among arabic, LBG and cherry gums as shown in the Fig. 1A–C. The gum arabic spectrum (Fig. 1A, Table 2, Supplementary Table 2) is characterized by trisaccharides (e.g. PentHex_2_) and tetrasaccharides (e.g. Pent_2_Hex_2_), as well as more complex monosaccharide combinations (e.g. PentHex_2_dHexHexA) including up to 7 saccharidic residues (e.g. Pent_2_Hex_3_dHexHexA) (complementary MS/MS experiments described in the following section). According to the literature^12^, it could be concluded that Pent corresponds to l-Ara, Hex to d-Gal, dHex to l-Rha and HexA to d-GlcA. The chemical composition of gum arabic may vary according to different factors such as plant source and species, age of the tree, area of collection and climatic conditions^16^,^46^,^47^. We thus applied our analytical methodology to various *Acacia senegal* var. *senegal* raw samples in order to confirm the consistency of the MS profile of gum arabic. Three samples collected in Western and Southeast Sudan (1994, 1999) showed a reproducible MS profile (Table 2, Supplementary Fig. 2). An additional analysis of a 50-year-old commercial sample was also consistent with the gum arabic mass fingerprint (Table 2, Supplementary Fig. 3). The cherry gum spectrum is reported in Fig. 1C. Even if the spectrum is characterized by a low signal/noise, we were able to identify K^+^ cationized-oligosaccharides with Pent_x_Hex_y_ composition (Supplementary Table 3). This result was consistent with the data reported in the literature, according to which cherry gum is mainly composed of Ara (35.1%) and Gal (37.0%)^35^. The lower signal/noise of the cherry gum MS spectrum possibly can be due to both the limited access of the enzyme to the target backbone and the reduced solubility of the gum in our experimental conditions due, respectively, to the cherry gum structure and its various physicochemical properties. However, even if the proposed method seems less sensitive to cherry gum than to gum arabic, the MS profile is still distinguishable and cherry gum discrimination is possible. A very distinct pattern was also observed for LBG. The MS profile of digested LBG (Fig. 1B) resulted in a profile distinctly different from the ones obtained from the other arabinogalactan plant gums, mainly due to the absence of pentosyl residues. As for the cherry gum fingerprint, the K^+^ adducts dominated the mass spectrum but the corresponding oligosaccharides cationized with H^+^ and Na^+^ were also observed. Previous analysis by ESI-MS^n^ of LBG digested with endo-1,4-β-mannanase^28^ showed the presence of Hex_n_ oligosaccharides with n = 3–6. The mass spectrum obtained in this work was characterized by oligosaccharides composed of 3 up to 14 hexoses (Supplementary Table 4). It can be pointed out that several other gums commonly indicated as possible sources of polysaccharides in paint binders were also successfully tested with the enzyme cocktail^48^ such as gum tragacanth despite its complex composition (e.g. tragacanthin is water soluble, bassorin swells in water)^19^,^49^,^50^,^51^,^52^. The spectrum is characterized by intense peaks of oligosaccharides from four saccharidic residues up to seven with various composition such as Hex_2_HexA_2_, dHex_4_HexA_2_ or Pent_2_dHex_5_ (see details in experimental section, Supplementary Fig. 4 and Supplementary Table 5). It is therefore possible to conclude that the developed enzyme cocktail works optimally for both arabinogalactan-like gums (such as gum arabic, cherry gum), and for galactomannans polysaccharides (such as LBG) thus allowing a characteristic MS fingerprint for each natural polymer to be obtained from a mixture.

#### Characterization of saccharidic mass fingerprints using MALDI-TOF MS/MS experiments

**Figure 2:**
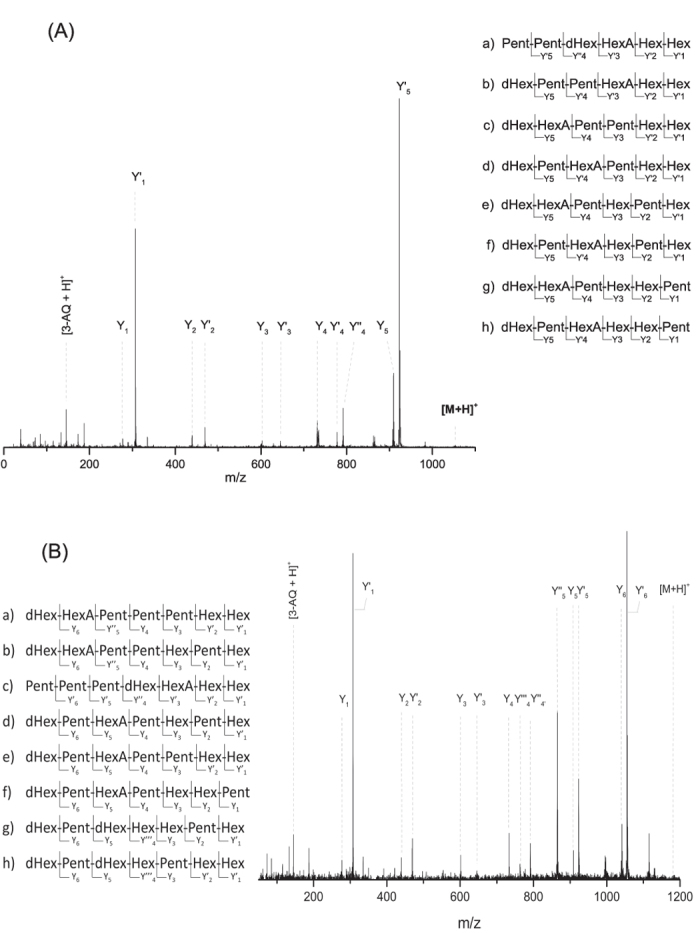
(**A**) MALDI-TOF MS/MS spectrum of the ion [3-AQ/M + H]^+^ at *m/z* 1055.360 from the digestion of gum arabic with the enzyme cocktail and list of the possible isomers of Pent_2_Hex_2_dHexHexA. The fragmented Y-ions (noted as Y, Y’ and Y” to distinguish the isomers) are observed. (**B**) MALDI-TOF MS/MS spectrum of the ion [3-AQ/M + H]^+^ at *m/z* 1187.409 from the digestion of gum arabic with the enzyme cocktail and list of the possible oligosaccharidic isomers of Pent_3_Hex_2_dHexHexA (a–f) and Pent_2_Hex_3_dHex_2_ (g,h). The fragmented Y-ions (noted as Y, Y’, Y” and Y”’ to distinguish the isomers) are observed. The y axis of spectra is the relative intensity.

With the aim of confirming the composition of the oligosaccharides enzymatically released from gum arabic, CID-fragmentation experiments of each ion observed in the MS spectrum were performed. Mass spectra were dominated by series of Y-ions (Fig. 2A, Supplementary Table 6) due to the proton affinity of quinoline (charge retention by the derivatized reducing end). Fragments were assigned using the Domon-Costello nomenclature^53^ and apostrophes (e.g. Y_4_’, Y_4_”, Y_4_”’) were used to distinguish the fragments ions from the various isomers. No ion resulting from cross-ring fragmentation was observed. For example, the Y-ions resulting from the fragmentation of the protonated ion at *m/z* 1055.360 (Fig. 2A) allowed to attribute the ion to the Pent_2_Hex_2_dHexHexA oligosaccharide. Based on the general monosaccharides occurring in AGs structure^12^, this oligosaccharide may correspond to Ara_2_Gal_2_RhaGlcA. Additionally, the presence of several isomers was identified: (a)-Pent_2_dHexHexAHex_2_ (Y’_5_Y”_4_Y’_3_Y’_2_Y’_1_); (b)-dHexPent_2_HexAHex_2_ (Y_5_Y’_4_Y’_3_Y’_2_Y’_1_); (c)-dHexHexAPent_2_Hex_2_ (Y_5_Y_4_Y_3_Y’_2_Y’_1_); (d)-dHexPentHexAPentHex_2_ (Y_5_Y’_4_Y_3_Y’_2_Y’_1_); (e)-dHexHexAPentHexPentHex (Y_5_Y_4_Y_3_Y_2_Y’_1_); (f)-dHexPentHexAHexPentHex (Y_5_Y’_4_Y_3_Y_2_Y’_1_); (g)-dHexHexAPentHex_2_Pent (Y_5_Y_4_Y_3_Y_2_Y_1_); and (h)-dHexPentHexAHex_2_Pent (Y_5_Y’_4_Y_3_Y_2_Y_1_) (Fig. 2A, Supplementary Table 6). To our knowledge the occurrence of different isomers has not been described for gum arabic but it has previously been reported in the case of enzymatic digestion of soybean pectic substances^54^. In comparison to the MS/MS profile of the ion at *m/*z 1055.360 (Fig. 2A), the fragmentation pattern of the protonated ion at *m/z* 1187.409 (Fig. 2B, Supplementary Table 6) showed the presence of two different oligosaccharides: Pent_3_Hex_2_dHexHexA (I) and Pent_2_Hex_3_dHex_2_ (II). Furthermore, several possible isomers were observed for each of the identified oligosaccharide, six for the oligosaccharide I (a–f) and two for the oligosaccharide II (g-h): (a)-dHexHexAPent_3_Hex_2_ (Y_6_Y”_5_Y_4_Y_3_Y’_2_Y’_1_); (b)-dHexHexAPent_2_HexPentHex (Y_6_Y”_5_Y_4_Y_3_Y_2_Y’_1_); (c)-Pent_3_dHexHexAHex_2_ (Y’_6_Y’_5_Y”_4_Y’_3_Y’_2_Y’_1_); (d)-dHexPentHexAPentHexPentHex (Y_6_Y_5_Y_4_Y_3_Y_2_Y’_1_), (e)-dHexPentHexAPent_2_Hex_2_ (Y_6_Y_5_Y_4_Y_3_Y’_2_Y’_1_); (f)-dHexPentHexAPentHex_2_Pent (Y_6_Y_5_Y_4_Y_3_Y_2_Y_1_); (g)-dHexPentdHexHex_2_PentHex (Y_6_Y_5_Y”’_4_Y_3_Y_2_Y’_1_); and (h)-dHexPentdHexHexPentHex_2_ (Y_6_Y_5_Y”’_4_Y_3_Y’_2_Y’_1_). Consequently, MS/MS analysis allowed the assignment of possible oligosaccharide compositions and it demonstrated the capability of identifying several isomers. Additionally, it can be pointed out that even if the gum arabic profile is not completely sequenced, the results obtained represent a great improvement in the understanding of the complex polysaccharide structure.

### Application of the developed method to real art samples

The optimized strategy was finally applied to the analysis of contemporary and precious historic watercolor samples, which are the main area of application of plant gums for artworks, and specifically of gum arabic. Beyond testing the analytical methodology for gum identification, the contemporary samples were selected in order to evaluate if the presence of other organic/inorganic materials could interfere with the MALDI-TOF analysis and, therefore, alter the gum mass profile. The contemporary watercolor artists’ tube paints n. 278 “Burnt Sienna” and n. 588 “Vermilion” were selected since the first has predominantly a mineral component (PBr7 calcined natural Sienna, an iron and manganese oxide) while the second one is characterized by the presence of organic colorants (diarylamide orange P034 and Naphthol red AS-OL PR9); both are described to contain arabic gum, preservatives, wetting agents and extenders. Sample filtration on polyvinylidene difluoride filters was evaluated prior analysis to facilitate the removal of pigment particle residues, but most of the oligosaccharides were retained on the filter, thus preventing their identification. The established protocol, without any clean-up or filtering step, was found to be superior. Unlike the standard *Acacia senegal*, the mass spectra were dominated by Na^+^ and K^+^ ions (Supplementary Fig. 5). Even if several unidentified ions were present in both spectra and the signal-to-noise was lower than that of the standard gum, the ions corresponding to the gum arabic mass fingerprint were successfully observed for the two watercolors (Table 2).

**Figure 3:**
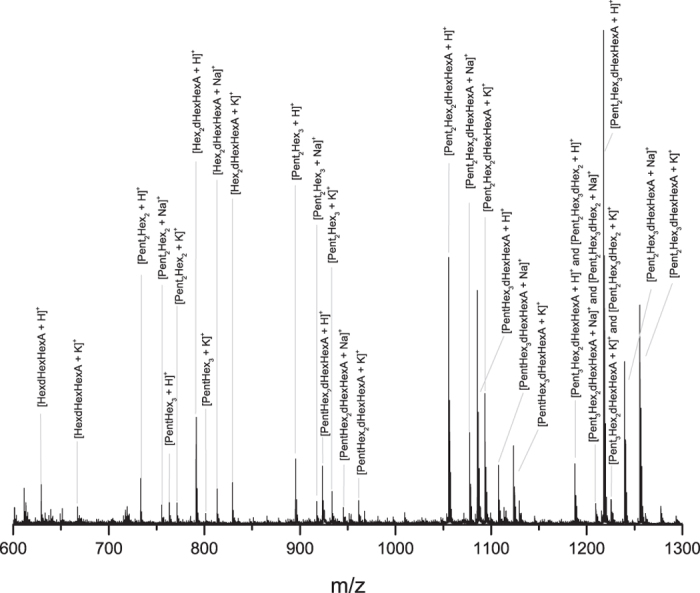
MALDI-TOF MS profile of the old watercolor sample dating 1870 with the related oligosaccharide attributions. The reported ions correspond to oligosaccharides derivatized with 3-aminoquinoline. The monosaccharide order for each oligosaccharide is arbitrary and does not refer to its structure. The mass spectrum is consistent with the oligosaccharidic fingerprint profile of *Acacia senegal* var. *senegal*. The y axis of the spectrum is the relative intensity.

Concerning the watercolor paint sample dating from 1870, Colour Box Charles Roberson & Co (Supplementary Fig. 1), little information was available about its composition and besides the blue pigment, the presence of gum arabic was presumed. X-ray fluorescence analysis (data not shown) showed the presence of cobalt (43%), thus indicating that the pigment is a cobalt-based blue, together with zinc (37.5%) and lead (15.5%), which could derive from the extenders. Fourier transform infrared spectroscopy (FTIR) was used as a first investigation of the organic binder (Supplementary Fig. 6). Results showed the potential presence of a polysaccharide-based material according to the band at 3383 cm^−1^ that can be assigned to OH stretching vibrations, 1605, 1415, 1072 and 667 cm^−1^. As shown in the Fig. 3 (Table 2, Supplementary Table 7), the intense signal to noise allowed the successful identification of the gum mass fingerprint and oligosaccharidic sequences. The protonated ions dominated the mass spectrum and Na^+^ and K^+^ adducts were also identified. The MALDI spectrum showed the presence of a wide *m/z* range of oligosaccharides corresponding to different Pent_x_Hex_y_dHex_z_HexA_k_ combinations, mostly with a degree of polymerization between 4 and 7 (e.g. Pent_2_Hex_2_dHexHexA). While only one trisaccharide was observed (HexdHexHexA), multiple tetrasaccharides were detected and they consisted of different combinations of pentosyl and hexosyl units (e.g. Pent_2_Hex_2_ and PentHex_3_), as well as hexosyl, deoxyhexosyl and hexuronosyl acids residues (Hex_2_dHexHexA). Most of the ions observed in the historic watercolor spectrum were attributed to a specific oligosaccharide and confirmed by MS/MS analysis. Results allowed to confirm the presence of gum arabic from *Acacia senegal* var. *senegal* in the 1870 Colour Box Charles Roberson & Co paint sample.

**Figure 4:**
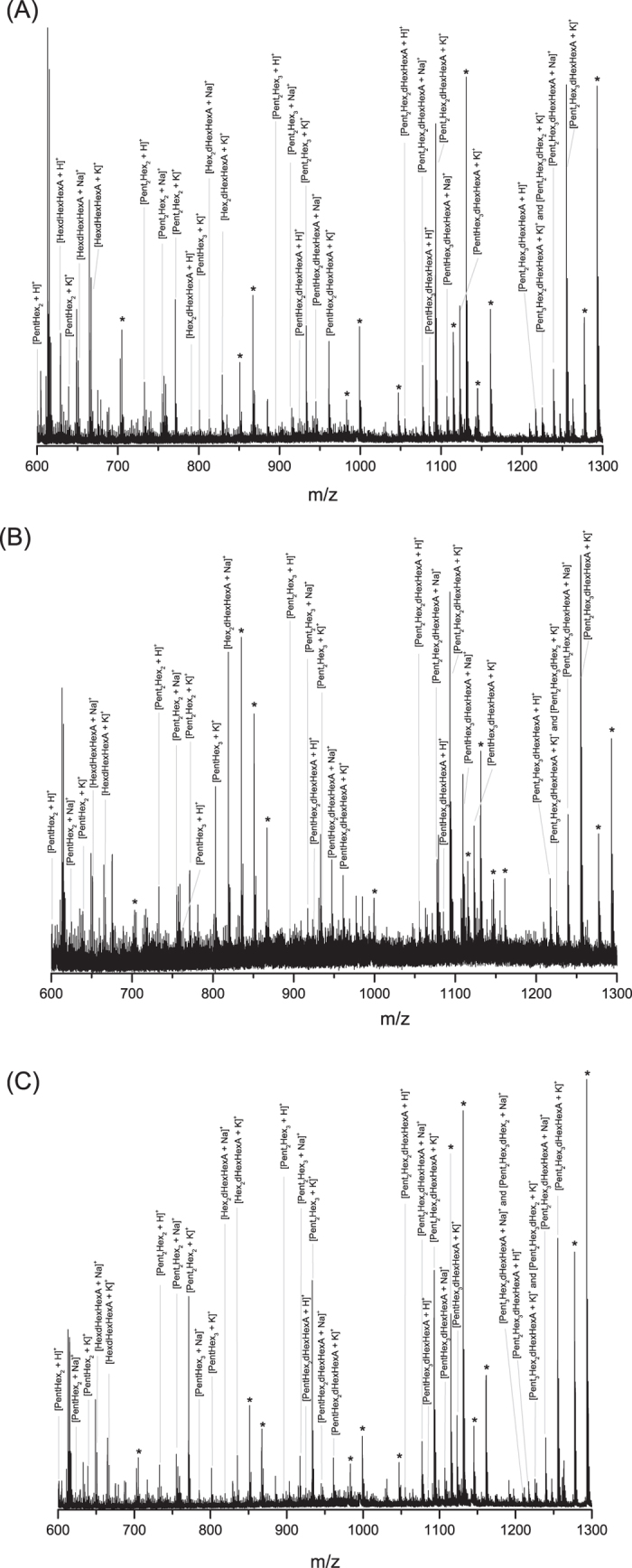
MALDI-TOF MS spectra of (**A**) the green and opaque sample (noted Dove 1) from *Boat houses* (Arthur Dove 1935, The Met #2005.484.3); (**B**) the grey sample (noted Dove 2) and (**C**) the light blue sample (noted Dove 3) from Study for ‘Goat’ (Arthur Dove 1934, The Met #49.70.75). *Not identified peaks or underivatized ions for *m/z* 983.306 (PentHex_5_ [M + Na]^+ *a*^), *m/z* 999.281 (PentHex_5_ [M + K]^+*b*^), *m/z* 1115.348 (Pent_2_Hex_5_ [M + Na]^+*c*^), *m/z* 1131.322 (Pent_2_Hex_5_ [M + K]^+*d*^), *m/z* 1277.401 (Pent_2_Hex_6_ [M + Na]^+*e*^) and *m/z* 1293.375 (Pent_2_Hex_6_ [M + K]^+*f*^). Derivatized forms of oligosaccharides^a,b,c,d,e,f^ are identified in the spectra: *a* at *m/z* 1109.364, *b* at *m/z* 1125.338, *c* at *m/z* 1241.406, *d* at *m/z* 1257.381, *e* at *m/z* 1403.459 (not shown), *f* at *m/z* 1419.459 (not shown).

Concerning the samples from Arthur Dove’s drawings, three samples were obtained from two drawings: one green and opaque sample (noted Dove 1 in Table 2) from *Boat Houses* (1935, The Metropolitan Museum of Art #2005.484.3) and two samples, a grey (noted Dove 2) and a light blue (noted Dove 3) ones, from *Study for ‘Goat’* (ca. 1934, The Metropolitan Museum of Art #49.70.75). The first drawing is described as ‘watercolor and ink on paper’, and the second as ‘watercolor, gouache, graphite on paperboard’, thus indicating the presence of different organic/inorganic materials. Arthur Dove is reported^55^ to have experimented prolifically by making his own paints in addition to artist’s tube paints. He is reported to have used a variety of binders (gum, egg, casein, oil, wax, resins, etc.), sometimes mixing them together, with pigments for specific passages. Because many of Dove’s works are on paper, sampling is extremely limited and there have been very few analytical studies of his paint binders. FTIR spectra showed the characteristic bands of a polysaccharide-based material, as previously observed with the watercolor paint sample from 1870. Besides these bands, FTIR showed the presence of an earth pigment in the sample Dove 1, while zinc white (ZnO) was observed by Raman in both samples Dove 2 and Dove 3. The total amounts of samples available (few tens of micrograms each) were used for MALDI-TOF analysis and results are shown in Fig. 4 and reported in Table 2. The K^+^ adducts dominated the mass spectrum and protonated and Na^+^ adducts were also identified. The limited amounts of available samples for analysis result in a lower signal-to-noise ratio of MS spectra (Fig. 4) compared to the ones of the previously described samples (e.g. Fig. 3 for 1870 Colour Box Charles Roberson & Co paint sample). Despite the presence of a few non attributed peaks or underivatized oligosaccharides, most of the ions observed in the three Dove’ samples in the m/z range between 600 and 1300 were successfully attributed to 3-AQ derivatized and specific oligosaccharides as a result of MS/MS analysis. The profiles of MS spectra composed by various degree of polymerization of Pent_x_Hex_y_dHex_z_HexA_k_ (e.g. Pent_2_Hex_3_dHexHexA for the most complex structure, PentHex_2_ for the simpler one) are similar to the arabic gum profile previously described and validate the use of MS oligosaccharide mass fingerprint for gum identification. For these three samples from Dove’s drawings, the use of *Acacia senegal* var. *senegal* is thus confirmed. The result revealed how the presence of pigment particles and other additives, together with the ageing, does not seem to significantly interfere with the analysis since the mass fingerprint of the gum is preserved.

## Discussion

A new method based on the partial enzymatic digestion of plant gum polysaccharides coupled to MALDI-TOF MS and MS/MS analysis was successfully developed to the identification of arabic, cherry and locust bean gums in various samples including samples from cultural heritage. The complete protocol takes less than 24 hours, a minimum sample manipulation is required and the strategy can be applied independently of the presence of gums’ mixtures thanks to the development of an enzyme cocktail. Analysis of raw plant gums demonstrated how the strategy developed on standard natural polysaccharides is applicable also to more complex and partially unknown structures. We have provided evidence that the MS spectra obtained for gum arabic, cherry and locust bean gum (Fig. 1) are completely different and represent a specific fingerprint of each gum, thus allowing their discrimination. MS/MS experiments allowed the accurate identification of the oligosaccharidic chains and revealed the presence of multiple isomers (Fig. 2); this shows the power of mass spectrometry in exposing the structure of complex compounds. The mass profile of gum arabic was confirmed by analyzing several *Acacia senegal* var. *senegal* samples from different ages and geographical origins as well as more complex samples, i.e. commercial watercolors. In all cases, starting from low sample amounts, results showed the presence of gum arabic, thus demonstrating that ageing, as well as the possible presence of other organic/inorganic components, does not cause any significant alteration of the gum MS profile. Finally, the strategy was successfully applied to the analysis of 1 mg watercolor sample from a colour box dating 1870 and few micrograms of three samples from Arthur Dove’s drawings dating 1934–1935 (*Boat houses* and *Study for ‘Goat’*) from the Metropolitan Museum of Art. The obtained spectra have exhibited the almost complete mass fingerprint of the gum arabic, allowing thus its successful identification. In the cultural heritage field, establishing the gum identification based on the oligosaccharide mass fingerprint, and not on the monosaccharide’s proportions by GC-MS, is much more informative since each ion represents an accurate oligosaccharide that belongs to a specific gum. In this way misidentification of gums is prevented. The possibility of distinguishing different gums in a mixture, the easy sample preparation protocol and the short-timing strategy, represent a significant improvement in the field of conservation science. The method suggested in this paper is strong and represents a promising and powerful new strategy for plant gums identification in complex samples. This is the first time that gum is accurately identified in historic cultural heritage samples using structural information. It can be pointed out that this straightforward methodology is applicable to industries (food/cosmetic/pharmaceutical/biomedical).

## Methods

### Reagents and enzymes

α-l-Arabinofuranosidase (EC 3.2.1.55), exo-β-1,3-galactanase (EC 3.2.1.145), endo-β-1,4-mannanase (EC 3.2.1.78) and α-galactosidase (EC. 3.2.1.22) were purchased from NZYtech (Lisboa, Portugal). β-Galactosidase (EC 3.2.1.23), β-glucuronidase (EC 3.2.1.31), and α-l-rhamnosidase (EC 3.2.1.40) were purchased from Megazime (Wicklow, Ireland). Larch wood arabinogalactan polysaccharide and carob galactomannan were obtained from Megazyme (Wicklow, Ireland). Deionized water was obtained from Millipore cartridge equipment (Bedford, MA). All other solvents and chemicals were obtained from Sigma Aldrich (L’lsle d’Abeau Chesnes, France).

### Gum and commercial watercolor samples

Locust bean gum was purchased from Sigma Aldrich (L’lsle d’Abeau Chesnes, France). Cherry gum, was collected in the Veneto region (Italy). The standard gum arabic from *Acacia senegal* var. *senegal* was obtained from Zecchi (Florence, Italy). The other gum arabic samples from *Acacia senegal* var. *senegal* were kindly supplied by Prof. P. C. Ravines (Director of Buffalo State’s renowned Art Conservation Department, Buffalo State University – School of Arts and Humanities). All the three samples were collected in Sudan. The first sample is from Kordufan (Western Sudan) and it was collected in 1994. The two other samples were collected in Damazene (Southeast Sudan) in 1994 and 1999. The contemporary watercolors were purchased from Maimeri (Mediglia, Italy) and Daler-Rowney (Bracknell, England). The watercolor from Maimeri is Burnt Sienna (n. 278); its composition provided by the manufacturer is gum arabic (Kurdufan, Sudan), glycerin and mineral pigment (PBr7 calcined natural Sienna). The watercolor from Daler-Rowney is Vermilion (n. 588); its composition provided by the manufacturer is gum arabic, glycerin, extenders, water, wetting agents, preservatives and pigment (diarylamide orange P034 and Naphtol red AS-OL PR9). The 50-year-old commercial gum arabic (*Acacia senegal* var. *senegal*) was provided by Rachel Mustalish (Department of Paper Conservation, The Metropolitan Museum of Art). The sample is a Winsor and Newton product. It was made in England in the 1960s and sold in the USA.

### Description of the Colour Box Charles Roberson & Co and Arthur Dove’s samples

The blue watercolor sample was obtained from a wooden artists’ paint box ‘Colour Box Charles Roberson & Co’ (see Supplementary Fig. 1) dating 1870s. The sample was supplied by Rachel Mustalish (Department of Paper Conservation, The Metropolitan Museum of Art). The box contains ten nearly intact watercolor pans and a set of three brushes, manually prepared, with a wooden stick and a feather quill to attach the bristles to the stick. Approximately 1 mg was sampled from the blue watercolor localized on the left in the box (noted “French Blue”). FTIR analysis was performed with a Hyperion 3000 FTIR spectrometer equipped with an IR 15x objective and a mercury cadmium telluride (MCT) detector. Analysis of the sample was run in transmission mode by flattening the sample in a diamond cell. Acquisitions were performed in the range of 4000–600 cm^−1^ at a resolution of 4 cm^−1^. Each spectrum is the sum of several scans (128–256) according to the response of the different samples. Library databases (MMA and IRUG Edition 2000 and 2007) were used for spectra interpretations. Three samples were obtained from two drawings by Arthur Dove (American, 1880–1946) in the collection of The Metropolitan Museum of Art: one green sample (Dove1) from *Boat* *Houses* (1935, The Metropolitan Museum of Art #2005.484.3) and two samples, a grey (Dove2) and a light blue (Dove3) ones, from Study for ‘Goat’ (ca. 1934, The Metropolitan Museum of Art #49.70.75). The first drawing is described as ‘watercolor and ink on paper’, and the second as ‘watercolor, gouache, graphite on paperboard’, thus indicating the presence of different organic/inorganic materials.

### Preparation of the polysaccharide-based samples

Larch wood arabinogalactan polysaccharide was prepared at 1% w/v in deionized water and the solution was stirred on a hot-plate magnetic stirrer at 80 °C until it was clear. The polysaccharide solution was kept at 4 °C until use. Carob galactomannan low viscosity and locust bean gum were wet with 95% MeOH (1 μL/mg of gum) and prepared at 1% w/v in MilliQ water. The solution was gently stirred for 1 hour and then left over night at 4 °C. The solution was then stirred on a hot-plate magnetic stirrer at 120 °C for 20 minutes. The supernatant, consisting of the soluble component of the gum, was recovered after centrifugation at 13400 rpm for 30 minutes. Standard gum arabic from *Acacia senegal* (powder) was solubilized in deionized water at a concentration of 1% w/v, mixed initially for 2 hours at room temperature and then over night at 4 °C. The same sample preparation procedure was employed for the preparation of A*cacia senegal* var. *senegal* nodules, which were collected in different areas of Sudan and of the watercolors samples (commercial and historic samples). In the case of turbid solution, due to the presence of pigment particles or other residues, the sample was centrifuged (13400 rpm, 20 minutes, repeated 4–5 times as necessary) until the solution was clear. Cherry gum was prepared according to the same procedure used for gum arabic and centrifuged at 13400 rpm for 30 minutes to collect the supernatant. Tragacanth gum (powder) was wet with 95% ethanol (1 μL/mg of gum) before the addition of deionized water to obtain a final concentration of 0.5% w/v. The solution was mixed initially for 2 hours at room temperature, then over night at 4 °C and finally centrifuged at 13400 rpm for 30 minutes to collect the supernatant.

### Enzymatic hydrolysis

An aliquot of each solution, corresponding to around 1 mg of polysaccharide, was collected, dried in a vacuum centrifuge at 30 °C (concentrator 5301, Eppendorf, Hamburg, Germany) and subjected to enzymatic digestion. If samples were not subjected immediately to digestion, they were dried and kept at −20 °C. The dried polysaccharide sample was resolubilized in 100 μL of the proper buffer, mixed vigorously and incubated with 100 mU of the selected enzyme for 5 hours. The following buffers and digestion temperatures were used (Table 1): 50 mM phosphate buffer pH 7, 65 °C; 100 mM phosphate buffer pH 6.8, 37 °C; 20 mM phosphate buffer pH 7, 45 °C; 50 mM acetate buffer pH 4.5, 60 °C, 50 mM phosphate buffer pH 6, 50 °C, 50 mM phosphate buffer pH 7.5, 45 °C and 25 mM Tris-HCl buffer, pH 8.5, 37 °C for respectively α-l-arabinofuranosidase (EC 3.2.1.55), β-glucuronidase (EC 3.2.1.31), α-l-rhamnosidase (EC 3.2.1.40), β-galactosidase (EC 3.2.1.23), exo-β-1,3-galactanase (EC 3.2.1.145), endo-β-1,4-mannanase (EC 3.2.1.78) and α-galactosidase (EC. 3.2.1.22). The enzymes α-L-arabinofuranosidase, β-glucuronidase and α-L-rhamnosidase were used at the beginning of the digestion in order to remove the terminus of the side chains and to de-branch the arabinogalactan structure. The action of the different enzymes on the gum sequence was evaluated using MALDI-TOF analysis (data not shown).

#### Hydrolysis of arabinogalactan standard polysaccharide

As a first experiment the standard arabinogalactan was hydrolyzed sequentially with the five enzymes in the corresponding order: α-l-arabinofuranosidase, β-glucuronidase, α-l-rhamnosidase, β-galactosidase and exo-β-1,3-galactanase. 1 mg of dried polysaccharide was solubilized in 100 μL of 50 mM phosphate buffer (pH 7) and 100 mU of α-l-arabinofuranosidase were added. The sample was incubated at 65 °C for 24 hours. Digestion was quenched by heating the sample solution at 100 °C for 15 minutes and the resulting solution was dried in a vacuum centrifuge at 30 °C. 100 μL of 100 mM phosphate buffer (pH 6.8) were successively added and the sample was incubated at 37 °C with 100 mU of the second enzyme β-glucuronidase for 24 hours. Digestion was quenched and all the other enzymes were added following the same procedure. The type of buffer, pH and temperature of incubation of each enzyme is reported in Table 1. After several tests, regarding both the digestion timing and the enzymes order and activities (25, 100, 500 and 1000 mU were evaluated), the best hydrolysis procedure for the standard arabinogalactan consisted in solubilizing the dried polysaccharide in 100 μL of 50 mM phosphate buffer, pH 6, and digesting it only with 100 mU of exo-β-1,3-galactanase for 24 hours at 50 °C.

#### Hydrolysis of galactomannan standard polysaccharide

As a first experiment the standard galactomannan was digested sequentially with α-galactosidase and endo-1,4-β-mannanases. 1 mg of dried polysaccharide was recovered in 100 μL of 25 mM Tris-HCl buffer (pH 8.5) and 100 mU of α-galactosidase were added. The sample was incubated at 37 °C for 5 hours. Digestion was quenched by heating the sample solution at 100 °C for 15 minutes and the resulting solution was dried in a vacuum centrifuge at 30 °C. 100 μL of 50 mM phosphate buffer (pH 7.5) were successively added and the sample was incubated at 45 °C with 100 mU of the second enzyme endo-β-1,4-mannanase for 5 hours. Digestion was quenched by heating the sample solution at 100 °C for 15 minutes. The solution was then dried in a vacuum centrifuge at 30 °C. After proper developments (including the evaluation of 25, 100, 500 and 1000 mU of enzymes) the best digestion procedure for galactomannan consisted in digesting the polysaccharide only with 100 mU of endo-β-1,4-mannanase.

#### Hydrolysis of gum arabic

As for the standard larch arabinogalactan, the following enzymes have been used in the listed order: (1) α-L-arabinofuranosidase; (2) β-glucuronidase; (3) α-L-rhamnosidase; (4) β-galactosidase; (5) exo-β-1,3-galactanase. 1 mg of gum arabic was initially digested with α-L-arabinofuranosidase for 24 hours, reaction was quenched by boiling the solution and an aliquot was collected and analyzed by MALDI-TOF. The left sample was dried, re-solubilized in the proper buffer and incubated for 24 hours with the second enzyme (β-glucuronidase). The same procedure was carried out for all the further enzymes (α-L-rhamnosidase, β-galactosidase) until exo-β-1,3-galactanase that was incubated for 3, 5 and 93 hours. Some peaks corresponding to oligosaccharides were observed in the mass spectrum only after the addition of the last enzyme exo-β-1,3-galactanase. Using 5 enzymes, the complete sample preparation lasted 1 week. A less number of enzymes was tested and combined in order to shorten the digestion time (not detailed here). As already tested on the standard larch arabinogalactan, among the different experiments a test was performed incubating the sample only with exo-β-1,3-galactanase for different time (5 minutes, 30 minutes, 1, 3, 5, 8, 24, 96, 120 hours, 1 week, 2 weeks, 3 weeks) and various enzyme amounts (25, 100, 500 and 1000 mU). In all the mass spectra it was possible to observe, in the mass range between 1000 and 1300 Da, the same ions obtained using five enzymes. Digestion already began after 5 minutes incubation but no oligosaccharides with a molecular weight over 1300 Da were observed. After 3, and mainly 5 hours, higher molecular weight oligosaccharides were released. As additional experiment, since the activity of the enzymes decreases with the time, more enzyme (with same activity than initial enzyme addition) was added every 1–3 days but no differences were observed.

Further experiments of gum hydrolysis with α-L-arabinofuranosidase and then exo-β-1,3-galactanase were run since, according to theory, the exo-Gal action should be impeded by the presence of side chains. No differences were observed between the mass spectra of the gum digested with the two enzymes (α-L-arabinofuranosidase + exo-β-1,3-galactanase) and only exo-β-1,3-galactanase. At the light of these considerations a final digestion protocol of gum arabic was established: 1 mg of polysaccharides is resuspended in a phosphate buffer 50 mM pH 6 and incubated with 100 mU of exo-β-1,3-galactanase for 5 hours at 50 °C.

#### Hydrolysis of cherry gum

α-L-arabinofuranosidase, β-glucuronidase, exo-β-1,3-galactanase and β-galactosidase (25, 100, 500 and 1000 mU evaluated) were tested on a sample of cherry gum. Dried gum was initially digested with α-l-arabinofuranosidase for 24 hours, reaction was quenched by boiling the solution and an aliquot was collected and analyzed by MALDI-TOF. The left sample was dried, re-solubilized in the proper buffer and incubated for 24 hours with the second enzyme etc. Each enzyme was incubated for 24 hours and different enzyme combinations were tested. Among all the different experiments only digestion performed with exo-β-1,3-galactanase led to positive and informative results. No differences were observed if the sample was incubated for 5, 24 or 48 hours, so the shortest time digestion was preferred. The final optimized protocol is: 1 mg of dried gum was solubilized in 100 μL of 50 mM phosphate buffer (pH 6) and incubated with 100 mU of exo-β-1,3-galactanase at 50 °C for 5 hours. Digestion was quenched by heating the sample solution at 100 °C for 15 minutes. The solution was then dried in a vacuum centrifuge at 30 °C.

#### Hydrolysis of locust bean gum

After digestion with α-galactosidase for 24 hours, the sample was incubated with endo-1,4-β-mannanase and aliquots were collected after 30 minutes, 1, 3, 8, 24 hours and 6 days. MALDI-TOF results for a digestion time over 5 h show few oligosaccharides in the mass range over 2000 Da in comparison to a shorter digestion time. It is therefore possible that α-galactosidase significantly facilitate the access of mannanase enzyme to the backbone, therefore increasing the extent of hydrolysis. However, the objective is not to digest completely the gum into monosaccharides, but to obtain a certain number of oligosaccharides in the mass spectrum that allow to define the gum mass fingerprint. For this reason, a simpler incubation using only endo-1,4-β-mannanase was preferred. Consequently, the optimized protocol is: 1 mg of dried gum was solubilized in 100 μL of 50 mM phosphate buffer (pH 7.5) and incubated at 45 °C with 100 mU of endo-β-1,4-mannanase for 5 hours. Digestion was quenched by heating the sample solution at 100 °C for 15 minutes. The solution was then dried in a vacuum centrifuge at 30 °C.

#### Hydrolysis of tragacanth gum

α-L-arabinofuranosidase, exo-β-1,3-galactanase and β-galactosidase (25, 100, 500 and 1000 mU evaluated) were tested on a sample of tragacanth gum. Dried gum was initially digested with α- L-arabinofuranosidase for 24 hours, reaction was quenched by boiling the solution and an aliquot was collected and analyzed by MALDI-TOF. The remaining sample was dried, re-solubilized in the proper buffer and incubated for 24 hours with the second enzyme etc. Each enzyme was incubated for 24 hours and different enzyme combinations were tested. Among all of the different experiments only digestion performed with exo-β-1,3-galactanase led to positive and informative results. No differences were observed if the sample was incubated for 5, 24 or 48 hours, so the shortest digestion time was preferred. The final optimized protocol is: 1 mg of dried gum was solubilized in 100 μL of 50 mM phosphate buffer (pH 6) and incubated with 100 mU of exo-β-1,3-galactanase at 50 °C for 5 hours. Digestion was quenched by heating the sample solution at 100 °C for 15 minutes. The solution was then dried in a vacuum centrifuge at 30 °C.

#### Enzyme cocktail and hydrolysis of a sample with unknown polysaccharidic composition: hydrolyis of the samples from the Colour Box Charles Roberson & Co and from drawings by Arthur Dove

Based on the protocol optimization described in the previous sections, the enzyme cocktail was prepared by mixing an equal amount of exo-β-1,3-galactanase and 1,4-β-mannanase in 35 mM NaHepes pH 7.5, 750 mM NaCl, 200 mM imidazole, 3.5 mM CaCl_2_, 0.02% sodium azide, 25% glycerol (Table 1). This enzyme cocktail was first successfully evaluated on the standard samples (galactomannans and type II AGs) and on the raw gum samples (arabic, cherry, locust bean gums) and identical spectra profiles to those preliminary obtained with one enzyme were achieved (data not shown). The hydrolysis experiment was then performed on approximately 1 mg of the commercial watercolors and on the historic watercolor sample. The samples were solubilized accordingly to the procedure previously described in the “preparation of the polysaccharide-based samples” section. Briefly, the sample was solubilized in deionized water (200 μl), mixed for 2 hours at room temperature and then over night at 4 °C. The sample was then centrifuged at 13400 rpm for 20 minutes and the centrifugation of the supernatant was repeated 5 times (until the solution was clear). The filtration of commercial watercolor on a polyvinylidene difluoride (PVDF) filter (0.22 μm diameter) was evaluated but it has resulted in the loss of oligosaccharides so it was not used in this study. The solution (water-solubilized commercial watercolors and water-solubilized historic watercolor) was dried in a vacuum centrifuge at 30 °C. The dried sample was resuspended in 100 μL of 50 mM phosphate buffer (pH 7) and incubated for 5 hours at 45 °C simultaneously with 100 mU of exo-β-1,3-galactanase and 1,4-β-mannanase. The final digested sample was dried in a vacuum centrifuge at 30 °C before analysis. Enzymatic digestion of the three samples from the drawings by Arthur Dove was performed directly on the samples without previous extraction of the polysaccharidic material due to the limited amount of material available (few tens of micrograms). After incubation (solution described previously) the sample was centrifuged (13400 rpm, 20 minutes), the supernatant was recovered, heated at 100 °C for 15 minutes, and dried in a vacuum centrifuge at 30 °C before analysis.

### Evaluation of desalting procedures

Two chromatographic phases were evaluated for sample desalting prior analysis: graphitized carbon (Hypersep Hypercarb zip tip; ThermoFisher Scientific) and HILIC (HyperSep HILIC 200 μl, ThermoFisher Scientific).

For the graphitized carbon phase, the sample was resuspended in MilliQ water containing 0.1% of trifluoroaetic acid (TFA). The tip (maximum volume 200 μL) was equilibrate with 2 mL of ACN 100% containing 0.1% TFA and then 2 mL of MilliQ water with 0.1% of TFA. The analytes were bound to the tip phase by aspirating and dispensing the sample 30–40 times. The phase was then washed with 2 mL of MilliQ water acidified with 0.1% FA and the oligosaccharides eluted with increasing percentage of ACN in MilliQ water acidified with 0.1% of TFA (0.1; 0.5; 1; 5; 10; 30; 50; 70; 100%). The samples are then dried in a vacuum centrifuge and resuspended in MilliQ water. The released oligosaccharides were not retained by the phase and they were eluted with the washing.

Regarding the HILIC protocol, the sample was resuspended in 200 μl of binding solution containing 15 mM ammonium acetate, pH 3.5, with 85% acetonitrile (ACN). The tip was wet using the releasing solution composed with 15 mM ammonium acetate, pH 3.5 with 5% ACN and the tip was washed with the binding solution. The analyte binding was performed and the sample was washed using the binding solution (200 μl for 10 times). Finally, the analyte was released using the releasing solution (200 μl) and the sample was dried in a vacuum centrifuge before analysis.

In both cases the sample was resuspended in 3 μl of water and analyzed by MALDI-TOF. Results showed no difference compared to the non desalted samples. Consequently, the MALDI-TOF MS and MS/MS analyses were performed in this work without preliminary desalting procedure.

### MALDI-MS/MS analysis

The dried digested sample was resuspended in 3 μL of deionized water and several dilutions were prepared (200–10 pmol). Derivatization and matrix preparation were performed according to a previously described protocol^45^. 3-Aminoquinoline (3-AQ) was prepared 20 mg/mL in ACN/water 1/2 (v/v) with 0.07% of inorganic acid to reach pH 5. 0.5 μL of sample were spotted on the MALDI target and 1 μL of 3-AQ solution was added. Matrix and sample were mixed 3 times on the MALDI target and left dried at room temperature for 1 hour. Analysis was carried out on a MALDI-TOF-TOF ABI 4800^+^ (AB Sciex, Ontario, Canada) equipped with a 200 Hz Nd-YAG laser of 355 nm wavelength. Spectra were obtained with a delayed extraction technology, in reflectron positive mode with a grid voltage of 16 kV and the extraction delay at 420 ns. A total of 3000 laser shots were accumulated for each spectrum. MS/MS analysis were performed in Collision-Induced Dissociation (CID) mode with air. The grid voltage was fixed at 7.3 kV and the collision cell at 7 kV. External calibration was performed using standards (Dextran) in the 600–1500 Da m/z range. Spectra were interpreted manually.

## Additional Information

**How to cite this article:** Granzotto, C. *et al*. Plant gum identification in historic artworks. *Sci. Rep.* **7**, 44538; doi: 10.1038/srep44538 (2017).

**Publisher's note:** Springer Nature remains neutral with regard to jurisdictional claims in published maps and institutional affiliations.

## Supplementary Material

Supplementary Files

## Figures and Tables

**Table 1:** Summary of enzymes used in this study and their action.

Enzyme (*source*)	Reference	Activity [U/mg]	Shipping buffer	Buffer, pH and T (°C) used in this study for the hydrolysis experiments*	Linkage hydrolysis**
α-L-arabinofuranosidase *Clostridium thermocellum*	EC 3.2.1.55	125	35 mM NaHepes pH 7.5, 750 mM NaCl, 200 mM imidazol, 3.5 mM CaCl_2_, 0.02% sodium azide, 25% glycerol.	Phosphate buffer 50 mM, pH 7, 65 °C.	Terminal non-reducing α-L-arabinofuranoside.
β-glucuronidase *Escherichia Coli*	EC 3.2.1.31	37000	20 mM Tris-HCl pH 7.5, 50 mM NaCl, 0.1 mM EDTA plus 0.02% (w/v) sodium azide.	Phosphate buffer 100 mM, pH 6.8, 37 °C.	Terminal non-reducing β-D-glucuronic acid residues.
α-L-rhamnosidase *Prokaryote*	EC 3.2.1.40	190	Ammonium sulphate suspension in 0.02% (w/v) sodium azide.	Phosphate buffer 20 mM, pH 6.5, 50 °C.	Terminal non-reducing α-L-rhamnose.
β-galactosidase *Aspergillus niger*	EC 3.2.1.23	175	3.2 M ammonium sulphate + 0.02% Na azide.	Acetate buffer 50 mM, pH 4.5, 60 °C.	Terminal non-reducing β-D-galactose.
exo-β-1,3-galactanase *Clostridium thermocellum*	EC 3.2.1.145	20	35 mM NaHepes pH 7.5, 750 mM NaCl, 200 mM imidazol, 3.5 mM CaCl_2_, 0.02% sodium azide, 25% glycerol	Phosphate buffer 50 mM, pH 6, 50 °C.	Terminal non-reducing β-D-galactose residues in β-1,3-galactan.
α-galactosidase *Cellvibrio mixtus*	EC. 3.2.1.22	150	35 mM NaHepes pH 7.5, 750 mM NaCl, 200 mM imidazol, 3.5 mM CaCl_2_, 0.02% sodium azide, 25% glycerol.	Tris-HCl buffer 25 mM, pH 8.5, 37 °C.	Terminal, non-reducing α-D-galactose.
endo-1,4-β-mannanase *Cellvibrio japonicus*	EC 3.2.1.78	2200	35 mM NaHepes pH 7.5, 750 mM NaCl, 200 mM imidazol, 3.5 mM CaCl_2_, 0.02% sodium azide, 25% glycerol.	Phosphate buffer 50 mM, pH 7.5, 45 °C.	(1–4)-β-D-Mannosidic linkages.
**Enzyme cocktail:** exo-β-1,3-galactanase *Clostridium thermocellum*/endo-1,4-β-mannanase *Cellvibrio japonicus* (v/v)	EC 3.2.1.145/EC 3.2.1.78	20/2200	35 mM NaHepes pH 7.5, 750 mM NaCl, 200 mM imidazol, 3.5 mM CaCl_2_, 0.02% sodium azide, 25% glycerol.	Phosphate buffer 50 mM, pH 7, 45 °C.	

^*^The enzymes’ activities were evaluated *via* experiments involving 25, 100, 500 and 1000 mU (see experimental part), the best results were obtained with 100 mU of exo-β-1,3-galactanase and 100 mU of endo-β-1,4-mannanase. The best incubation duration obtained in this study is 5 h.

^**^Information obtained by Megazyme (Wicklow, Ireland) and NZYtech (Lisboa, Portugal) suppliers, and by the *on-line* “The Comprehensive Enzyme Information System”, Braunschweig, Germany (http://www.brenda-enzymes.org/index.php4).

**Table 2:** List of the assigned oligosaccharides of the digested samples in this study (i.e. gum arabic mass fingerprint).

Theoretical mass [Da]^*^	Possible oligosaccharide*	Analyzed samples										
		A. Std sample	B. AS1 sample	C. AS2 sample	D. AS3 sample	E. n 278 sample	F. n 588 sample	G. 50-year old	H. 1870s sample	I. Dove 1	J. Dove 2	K. Dove 3
601.22	PentHex_2_ [3-AQ/M + H]^+^	X	—	X	X	**	**	X	—	X	X	X
623.21	PentHex_2_ [3-AQ/M+Na]^+^	X	—	—	—	**	**	X	—	—	X	X
629.22	HexdHexHexA [3-AQ/M+H]^+^	X	X	X	X	**	**	X	X	X	—	—
639.18	PentHex_2_ [3-AQ/M+K]^+^	X	—	—	—	**	**	X	—	X	X	X
651.20	HexdHexHexA [3-AQ/M+Na]^+^	X	X	X	X	**	**	X	—	X	X	X
667.18	HexdHexHexA [3-AQ/M+K]^+^	X	X	X	X	**	**	X	X	X	X	X
733.27	Pent_2_Hex_2_ [3-AQ/M+H]^+^	X	X	X	X	**	**	X	X	X	X	X
755.25	Pent_2_Hex_2_ [3-AQ/M+Na]^+^	X	X	X	X	**	**	X	X	X	X	X
763.28	PentHex_3_ [3-AQ/M+H]^+^	X	X	X	X	**	**	X	X	—	X	—
771.22	Pent_2_Hex_2_ [3-AQ/M+K]^+^	X	X	X	X	**	**	X	X	X	X	X
785.26	PentHex_3_ [3-AQ/M+Na]^+^	X	—	X	X	**	**	—	—	—	—	X
791.27	Hex_2_dHexHexA [3-AQ/M+H]^+^	X	X	X	X	**	**	X	X	X	—	—
801.23	PentHex_3_ [3-AQ/M+K]^+^	X	—	X	X	**	**	—		X	X	X
813.25	Hex_2_dHexHexA [3-AQ/M+Na]^+^	X	X	X	X	**	**	X	X	X	X	X
829.23	Hex_2_dHexHexA [3-AQ/M+K]^+^	X	X	X	X	**	**	X	X	X	—	X
895.32	Pent_2_Hex_3_ [3-AQ/M+H]^+^	X	X	X	X	X	X	X	X	X	X	X
917.30	Pent_2_Hex_3_ [3-AQ/M+Na]^+^	X	X	X	X	X	X	X	X	X	X	X
923.31	PentHex_2_dHexHexA [3-AQ/M+H]^+^	X	X	X	X	X	X	X	X	X	X	X
933.28	Pent_2_Hex_3_ [3-AQ/M+K]^+^	X	X	X	X	X	X	X	X	X	X	X
945.30	PentHex_2_dHexHexA [3-AQ/M+Na]^+^	X	X	X	X	X	X	X	X	X	X	X
961.27	PentHex_2_dHexHexA [3-AQ/M+K]^+^	X	X	X	X	X	X	X	X	X	X	X
1055.36	Pent_2_Hex_2_dHexHexA [3-AQ/M+H]^+^	X	X	X	X	X	X	X	X	X	X	X
1077.34	Pent_2_Hex_2_dHexHexA [3-AQ/M+Na]^+^	X	X	X	X	X	X	X	X	X	X	X
1085.37	PentHex_3_dHexHexA [3-AQ/M+H]^+^	X	X	X	X	X	X	X	X	X	X	X
1093.31	Pent_2_Hex_2_dHexHexA [3-AQ/M+K]^+^	X	X	X	X	X	X	X	X	X	X	X
1107.35	PentHex_3_dHexHexA [3-AQ/M+Na]^+^	X	X	X	X	X	X	X	X	X	X	X
1123.32	PentHex_3_dHexHexA [3-AQ/M+K]^+^	X	X	X	X	X	X	X	X	X	X	X
1187.40	Pent_3_Hex_2_dHexHexA [3-AQ/M+H]^+^	X	X	X	X	X	X	X	X	—	—	—
1187.43	Pent_2_Hex_3_dHex_2_ [3-AQ/M + H]^+^											
1209.38	Pent_3_Hex_2_dHexHexA [3-AQ/M + Na]^+^	X	X	X	X	X	X	X	X	X	X	X
1209.42	Pent_2_Hex_3_dHex_2_ [3-AQ/M + Na]^+^									X	—	X
1217.41	Pent_2_Hex_3_dHexHexA [3-AQ/M + H]^+^	X	X	X	X	X	X	X	X	X	X	X
1225.35	Pent_3_Hex_2_dHexHexA [3-AQ/M + K]^+^	X	X	X	X	X	X	X	X	X	X	X
1225.39	Pent_2_Hex_3_dHex_2_ [3-AQ/M + K]^+^									X	X	X
1239.39	Pent_2_Hex_3_dHexHexA [3-AQ/M + Na]^+^	X	X	X	X	X	X	X	X	X	X	X
1255.37	Pent_2_Hex_3_dHexHexA [3-AQ/M + K]^+^	X	X	X	X	X	X	X	X	X	X	X

A. standard sample from *Acacia senegal* var. *senegal* from Zecchi (Florence, Italy) (noted Std; related spectrum: Fig. 1A); B, C and D. *Acacia senegal* var. *senegal* AS1, AS2 and AS3 respectively collected in Kordufan (Western Sudan) in 1994, Damazene (Southeast Sudan) in 1999 and Damazene (Southeast wSudan) in 1994 (related spectra: Supplementary Fig. 2); E and F. contemporary watercolors purchased from Maimeri (Mediglia, Italy; sample n. 278) and Daler-Rowney (Bracknell, England; n. 588) (related spectra: Supplementary Fig. 5). G. The 50 year old gum arabic (*Acacia senegal* var. *Senegal*) (Winsor and Newton) (related spectrum: Supplementary Fig. 3); H. The blue watercolor sample from a wooden box ‘Colour Box Charles Roberson & Co’ dating 1870s (related spectra: Fig. 3). I. The green and opaque sample (noted Dove 1) from *Boat Houses* (Arthur Dove 1935, The Metropolitan Museum of Art #2005.484.3) (related spectrum: Fig. 4A); J. The grey sample (noted Dove 2) from Study for ‘Goat’ (Arthur Dove 1934, The Metropolitan Museum of Art #49.70.75) (related spectrum: Fig. 4B); K. The light blue sample (noted Dove 3) from Study for ‘Goat’ (Arthur Dove 1934, The Metropolitan Museum of Art #49.70.75) (related spectrum: Fig. 4C). The presence of the oligossacharides in the mass fingerprint of each sample is marked by X, the absence of the oligossacharides is noted -.

*Oligosaccharides are derivatized with 3-aminoquinoline (3-AQ) and charged with H^+^, Na^+^ or K^+^. The order of monosaccharides for each oligosaccharide is arbitrary and does not refer to its structure.

**Oligosaccharides detected but in a *m/z* range containing contaminations.
