# Supplementary material for: Plant gum identification in historic artworks
Source: Sci Rep. 2017 Apr 20;7:44538. doi: 10.1038/srep44538 (PMC5397872; doi:10.1038/srep44538)
Supplement: Supplementary Files [file srep44538-s1.pdf]

Plant gum identification in historic artworks

Clara Granzotto[1,2,4], Julie Arslanoglu[3], Christian Rolando[1], Caroline Tokarski[1,*]

[1] Miniaturisation pour l'Analyse, la Synthèse & la Protéomique (MSAP), USR CNRS 3290, Université de Lille 1 Sciences et Technologies, 59655 Villeneuve d'Ascq, France.

[2] Department of Molecular Sciences and Nanosystems, Ca' Foscari University, Venice, Italy.

[3] Department of Scientific Research, The Metropolitan Museum of Art, New York, NY, USA.

[4] Present address: Department of Scientific Research, The Metropolitan Museum of Art, New York, NY, USA.

* Correspondence has to be addressed to Caroline.Tokarski@univ-lille1.fr

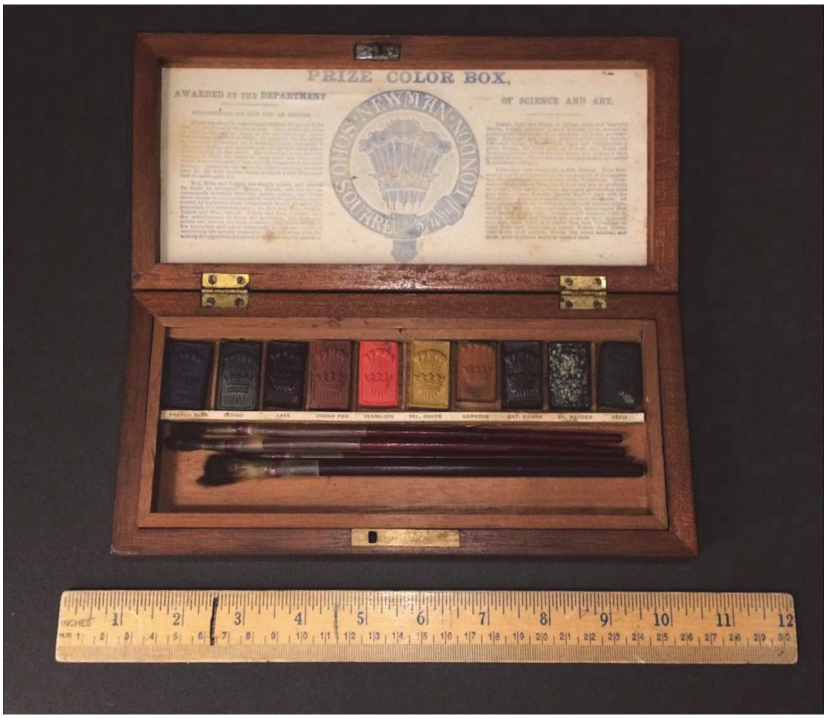

Supplementary Figure 1. Wooden 'Colour Box Charles Roberson & Co’ dating 1870s. The box includes watercolor pans and a set of three brushes, manually prepared, with a wooden stick and a feather quill to attach the bristles to the stick. Approximately 1 mg was sampled from the blue watercolor paint located on the left in the box (noted “French Blue”).

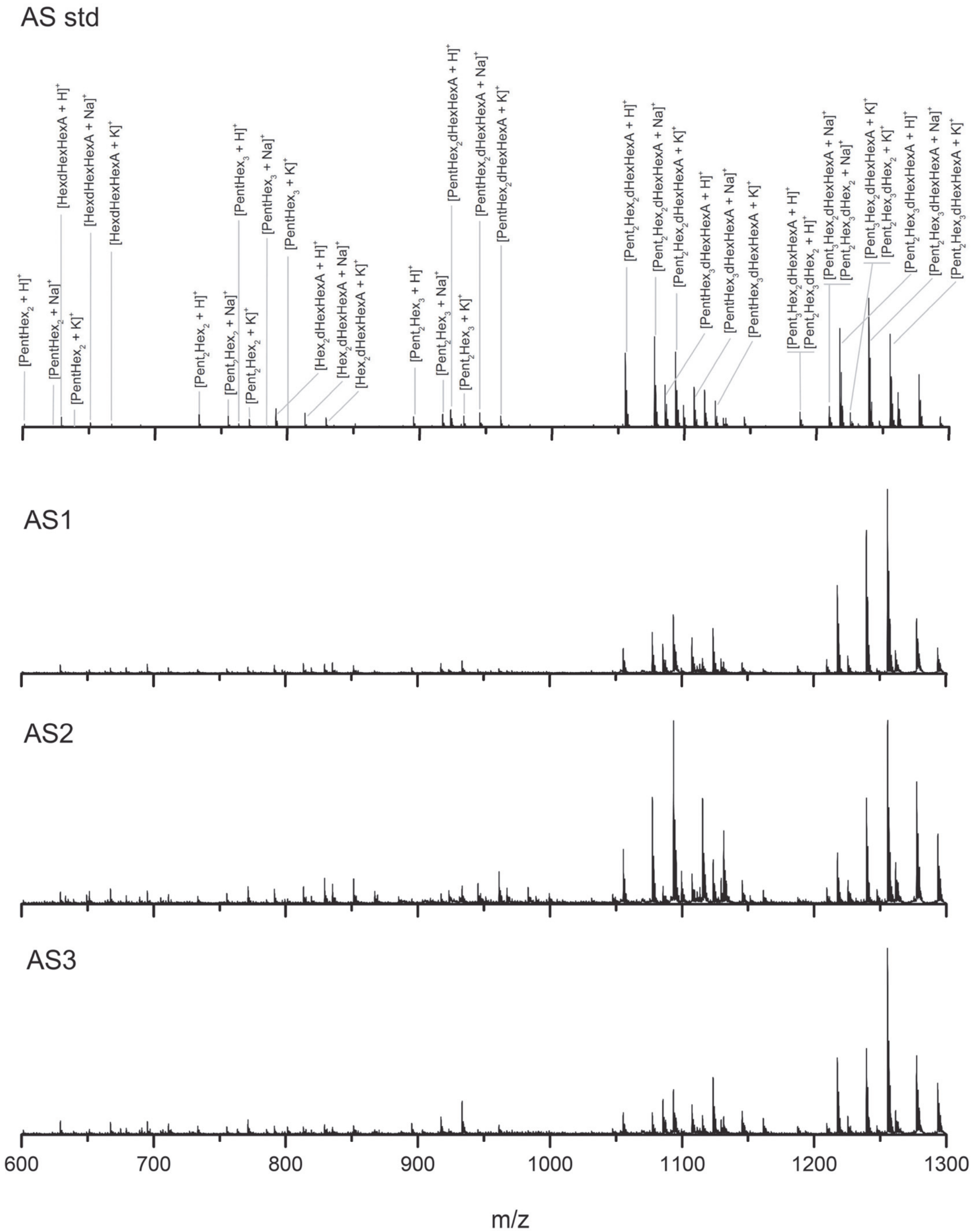

Supplementary Figure 2. MALDI-TOF MS spectra of digested *Acacia senegal* var. *senegal* samples (AS1, AS2 and AS3) compared to the standard gum arabic (AS std). *Acacia senegal* var. *senegal* AS1, AS2 and AS3 were respectively collected in Kordufan (Western Sudan) in 1994, Damazene (Southeast Sudan) in 1999 and Damazene (Southeast Sudan) in 1994. The y axis of spectra is the relative intensity.

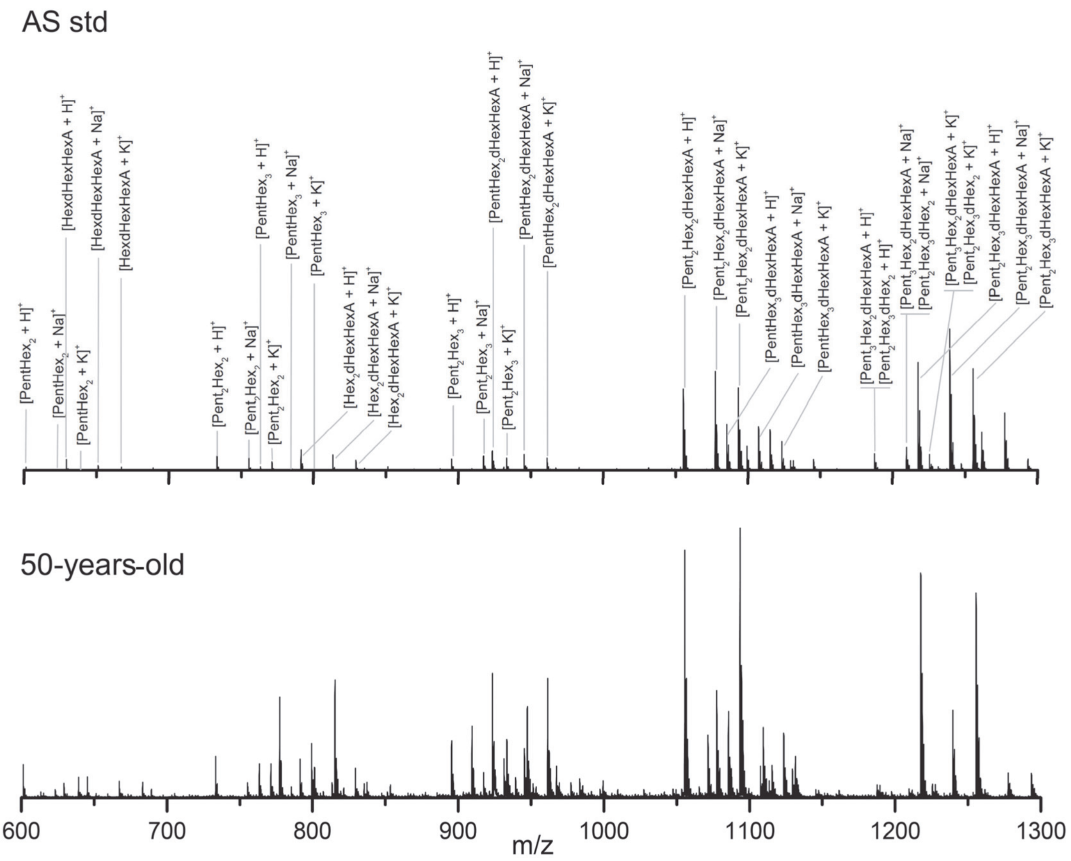

Supplementary Figure 3. MALDI-TOF MS spectra of digested 50-year-old gum arabic (The Metropolitan Museum of Art; supplier: Winsor and Newton) compared to the standard gum arabic (AS std). The y axis of the spectra is relative intensity.

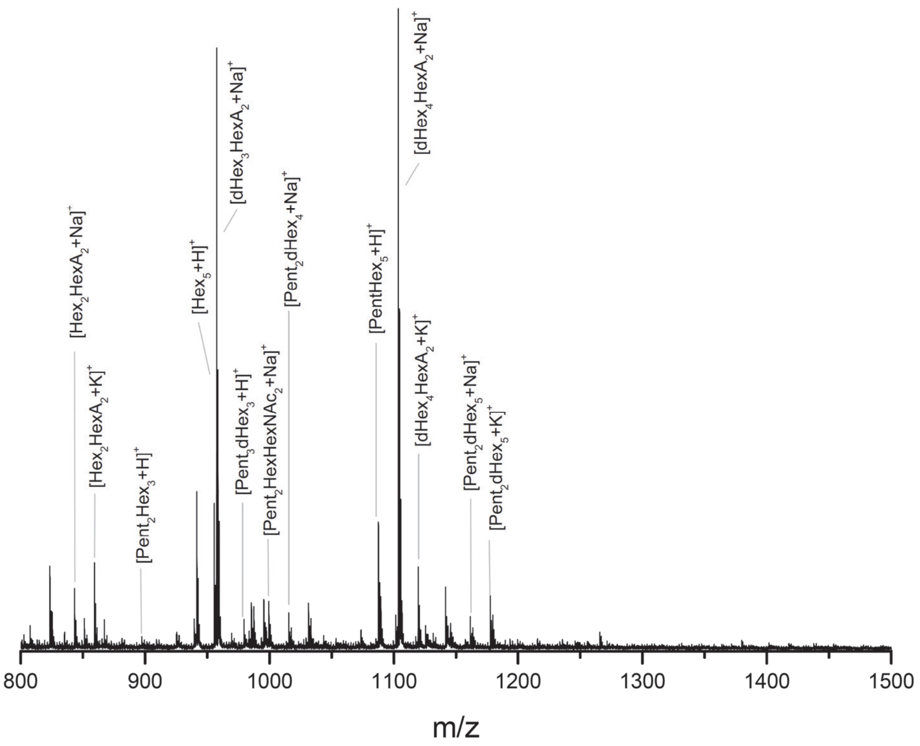

Supplementary Figure 4. MALDI-TOF MS spectrum of the enzymatically digested gum tragacanth. The monosaccharide order for each oligosaccharide is arbitrary and does not refer to its structure. The y axis of spectra is the relative intensity.

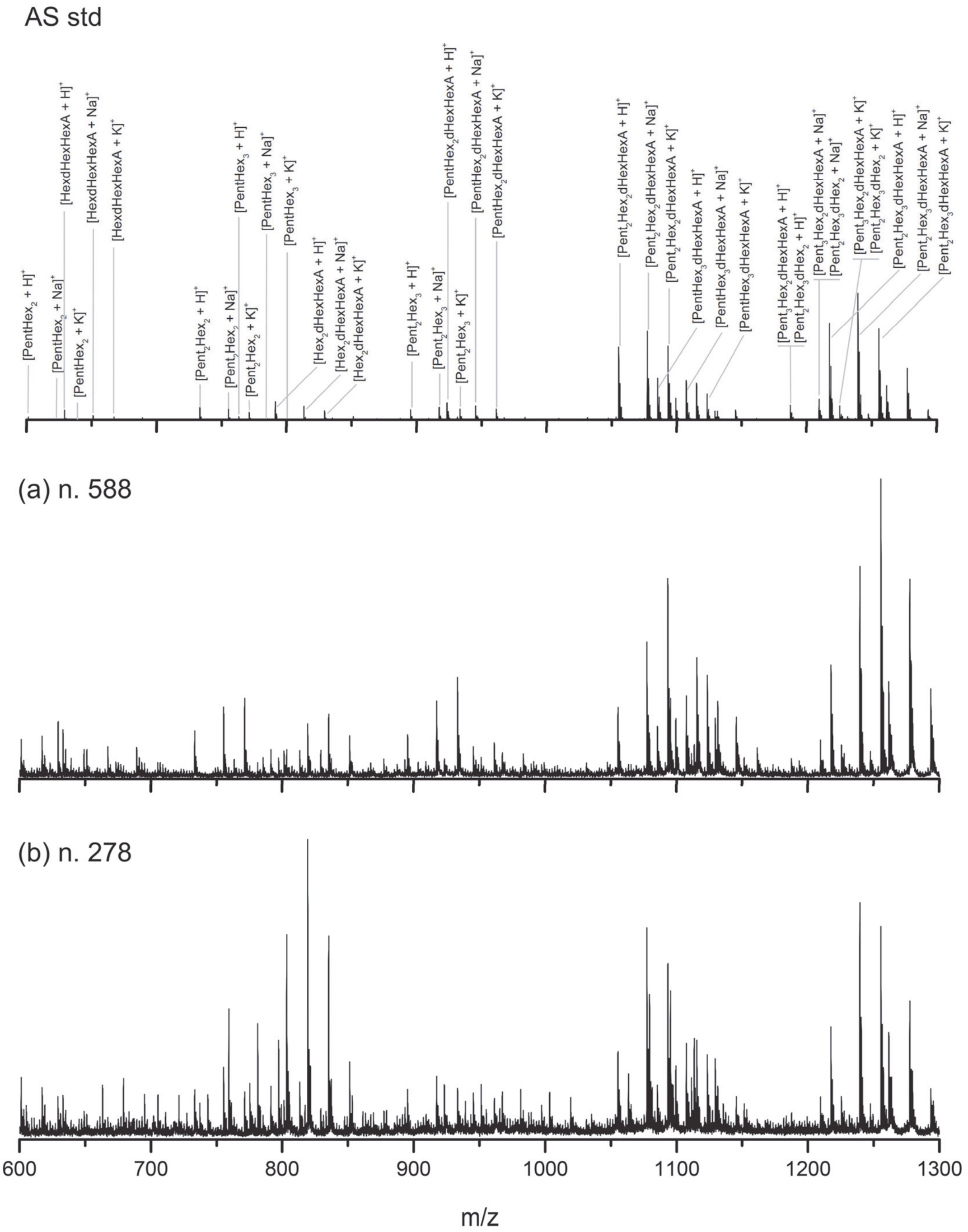

Supplementary Figure 5. MALDI-TOF MS spectra of contemporary watercolors sample n. 278 Burnt Sienna (Maimeri) and n. 588 Vermilion (Daler-Rowney) compared to the standard gum arabic (AS std). The y axis of spectra is the relative intensity.

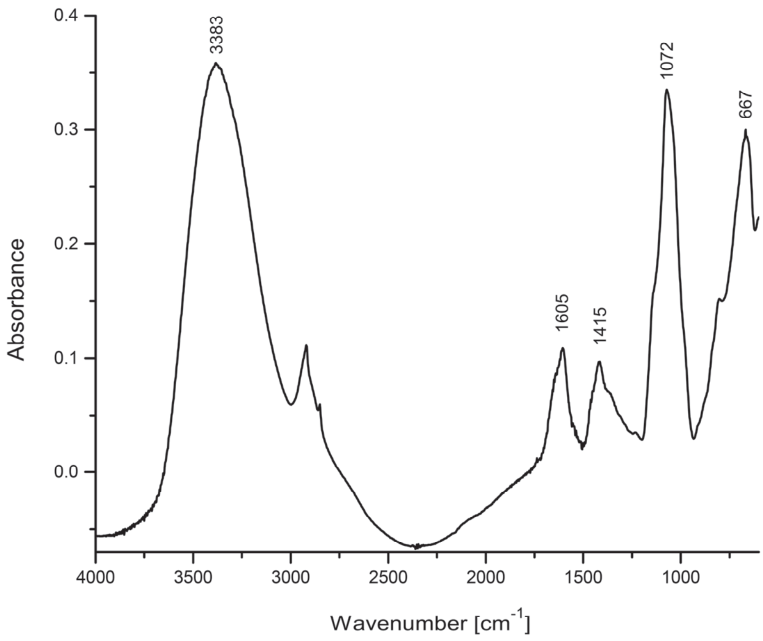

Supplementary Figure 6. FTIR spectrum of the French Blue paint from 'Colour Box Charles Roberson & Co' dating 1870s. The bands can be attributed to a polysaccharide based material: 3383 (from the OH stretching vibration), 1605, 1415, 1072 and 667 cm^{-1}.

Supplementary Table 1. List of monosaccharides, their respective abbreviation, structure, formula and monoisotopic mass.

Monosaccharide (abbreviation)	Structure (abbreviation)	Formula	Monoisotopic mass [Da]	
			Intact	Residue (intact - H_2O)
Pentose (Pent)	Arabinose (Ara) (Arabinofuranose, Ara*f* Arabinopyranose, Ara*p*)	$C_5H_{10}O_5$	150.05	132.04
Hexose (Hex)	Galactose (Gal) Mannose (Man) Glucose (Glu)	$C_6H_{12}O_6$	180.06	162.05
Deoxyhexose (dHex)	Rhamnose (Rha) Fucose (Fuc)	$C_6H_{12}O_5$	164.07	146.06
Hexuronic acid (HexA) (Uronic acid, UA)	Glucuronic acid (GlcA) Galacturonic acid (GalA)	$C_6H_{10}O_7$	194.04	176.03
N-Acetylamine hexose (HexNAc)	N-acetylgalactosamine (GalNAc)	$C_8H_{15}NO_6$	221.09	203.08
N-Acetylneuraminic acid (NeuAc) / Sialic acid (SA)		$C_{11}H_{19}NO_9$	309.11	291.09
N-Glycolyl neuraminic acid (NeuGc)		$C_{11}H_{19}NO_{10}$	325.10	307.09

Supplementary Table 2. List of the assigned oligosaccharides of the digested gum arabic corresponding to the **Fig. 1A** (i.e. gum arabic mass fingerprint).

Experimental mass [Da]	Δm [ppm]	Possible oligosaccharide*
601.219	- 2	$PentHex_2$ $[3\text{-AQ}/M+H]^+$
623.208	- 4	$PentHex_2$ $[3\text{-AQ}/M+Na]^+$
629.217	- 4	HexdHexHexA $[3\text{-AQ}/M+H]^+$
639.177	- 5	$PentHex_2$ $[3\text{-AQ}/M+K]^+$
651.196	- 6	HexdHexHexA $[3\text{-AQ}/M+Na]^+$
667.175	- 7	HexdHexHexA $[3\text{-AQ}/M+K]^+$
733.272	2	$Pent_2Hex_2$ $[3\text{-AQ}/M+H]^+$
755.250	0	$Pent_2Hex_2$ $[3\text{-AQ}/M+Na]^+$
763.280	0	$PentHex_3$ $[3\text{-AQ}/M+H]^+$
771.229	12	$Pent_2Hex_2$ $[3\text{-AQ}/M+K]^+$
785.258	- 2	$PentHex_3$ $[3\text{-AQ}/M+Na]^+$
791.278	10	$Hex_2dHexHexA$ $[3\text{-AQ}/M+H]^+$
801.227	- 3	$PentHex_3$ $[3\text{-AQ}/M+K]^+$
813.247	- 4	$Hex_2dHexHexA$ $[3\text{-AQ}/M+Na]^+$
829.226	- 5	$Hex_2dHexHexA$ $[3\text{-AQ}/M+K]^+$
895.311	- 10	$Pent_2Hex_3$ $[3\text{-AQ}/M+H]^+$
917.300	0	$Pent_2Hex_3$ $[3\text{-AQ}/M+Na]^+$
923.309	- 1	$PentHex_2dHexHexA$ $[3\text{-AQ}/M+H]^+$
933.279	- 1	$Pent_2Hex_3$ $[3\text{-AQ}/M+K]^+$
945.298	- 2	$PentHex_2dHexHexA$ $[3\text{-AQ}/M+Na]^+$
961.267	- 3	$PentHex_2dHexHexA$ $[3\text{-AQ}/M+K]^+$
1055.360	0	$Pent_2Hex_2dHexHexA$ $[3\text{-AQ}/M+H]^+$
1077.338	- 2	$Pent_2Hex_2dHexHexA$ $[3\text{-AQ}/M+Na]^+$
1085.378	7	$PentHex_3dHexHexA$ $[3\text{-AQ}/M+H]^+$
1093.317	6	$Pent_2Hex_2dHexHexA$ $[3\text{-AQ}/M+K]^+$
1107.346	- 4	$PentHex_3dHexHexA$ $[3\text{-AQ}/M+Na]^+$
1123.325	4	$PentHex_3dHexHexA$ $[3\text{-AQ}/M+K]^+$
1187.409	8	$Pent_3Hex_2dHexHexA$ $[3\text{-AQ}/M+H]^+$
1187.409	- 17	$Pent_2Hex_3dHex_2$ $[3\text{-AQ}/M+H]^+$
1209.398	14	$Pent_3Hex_2dHexHexA$ $[3\text{-AQ}/M+Na]^+$
1209.398	- 19	$Pent_2Hex_3dHex_2$ $[3\text{-AQ}/M+Na]^+$
1217.41	- 3	$Pent_2Hex_3dHexHexA$ $[3\text{-AQ}/M+H]^+$
1225.366	13	$Pent_3Hex_2dHexHexA$ $[3\text{-AQ}/M+K]^+$
1225.366	- 19	$Pent_2Hex_3dHex_2$ $[3\text{-AQ}/M+K]^+$
1239.385	- 4	$Pent_2Hex_3dHexHexA$ $[3\text{-AQ}/M+Na]^+$
1255.364	- 5	$Pent_2Hex_3dHexHexA$ $[3\text{-AQ}/M+K]^+$

* Oligosaccharides are derivatized with 3-aminoquinoline (3-AQ) and ionized with H^+. Na^+ or K^+. The order of monosaccharides for each oligosaccharide is arbitrary and does not refer to its structure.

Supplementary Table 3. List of the main oligosaccharides of the digested cherry gum corresponding to the **Fig. 1C** (i.e. cherry gum mass fingerprint).

Experimental mass [Da]	Δm [ppm]	Possible oligosaccharide*
771.218	- 2	$Pent_2Hex_2$ $[3\text{-AQ/M} + K]^+$
835.304	5	$Pent_2Hex_3$ $[3\text{-AQ/M} + K]^+$
933.298	19	$Pent_4Hex$ $[3\text{-AQ/M} + H]^+$
1095.336	5	$Pent_2Hex_4$ $[3\text{-AQ/M} + K]^+$
1227.365	- 4	$Pent_3Hex_4$ $[3\text{-AQ/M} + K]^+$
1359.413	- 5	$Pent_4Hex_4$ $[3\text{-AQ/M} + K]^+$
1491.441	- 6	$Pent_5Hex_4$ $[3\text{-AQ/M} + K]^+$
1623.497	- 2	$Pent_6Hex_4$ $[3\text{-AQ/M} + K]^+$
1755.533	- 4	$Pent_7Hex_4$ $[3\text{-AQ/M} + K]^+$
1887.568	- 7	$Pent_8Hex_4$ $[3\text{-AQ/M} + K]^+$
2019.612	- 4	$Pent_9Hex_4$ $[3\text{-AQ/M} + K]^+$

* Oligosaccharides are derivatized with 3-aminoquinoline (3-AQ) and ionized with H^+ or K^+. The order of monosaccharides for each oligosaccharide is arbitrary and does not refer to its structure.

Supplementary Table 4. List of the main oligosaccharides of the digested LBG corresponding to the **Fig. 1B** (i.e. LBG mass fingerprint).

Experimental mass [Da]	Δm [ppm]	Possible oligosaccharide*
631.227	- 4	Hex_3 [3-AQ/M + H]$^+$
653.216	- 6	Hex_3 [3-AQ/M + Na]$^+$
669.185	- 7	Hex_3 [3-AQ/M + K]$^+$
705.183	5	Hex_4 [M + K]$^+$
793.278	- 2	Hex_4 [3-AQ/M + H]$^+$
815.267	- 4	Hex_4 [3-AQ/M + Na]$^+$
831.336	- 5	Hex_4 [3-AQ/M + K]$^+$
867.233	- 8	Hex_5 [M + K]$^+$
955.337	- 3	Hex_5 [3-AQ/M + H]$^+$
977.316	- 4	Hex_5 [3-AQ/M + Na]$^+$
993.295	- 5	Hex_5 [3-AQ/M + K]$^+$
1029.292	2	Hex_6 [M + K]$^+$
1117.395	5	Hex_6 [3-AQ/M + H]$^+$
1139.374	3	Hex_6 [3-AQ/M + Na]$^+$
1155.352	2	Hex_6 [3-AQ/M + K]$^+$
1191.349	8	Hex_7 [M + K]$^+$
1279.442	2	Hex_7 [3-AQ/M + H]$^+$
1301.430	0	Hex_7 [3-AQ/M + Na]$^+$
1317.399	- 1	Hex_7 [3-AQ/M + K]$^+$
1353.405	4	Hex_8 [M + K]$^+$
1441.497	- 2	Hex_8 [3-AQ/M + H]$^+$
1463.475	- 3	Hex_8 [3-AQ/M + Na]$^+$
1479.453	2	Hex_8 [3-AQ/M + K]$^+$
1441.507	5	Hex_9 [M + K]$^+$
1603.541	- 6	Hex_9 [3-AQ/M + H]$^+$
1625.529	- 1	Hex_9 [3-AQ/M + Na]$^+$
1641.517	4	Hex_9 [3-AQ/M + K]$^+$
1677.493	- 4	Hex_{10} [M + K]$^+$
1765.593	- 4	Hex_{10} [3-AQ/M + H]$^+$
1787.591	0	Hex_{10} [3-AQ/M + Na]$^+$
1803.569	5	Hex_{10} [3-AQ/M + K]$^+$
1839.555	3	Hex_{11} [M + K]$^+$
1965.609	0	Hex_{11} [3-AQ/M + K]$^+$
2001.615	2	Hex_{12} [M + K]$^+$
2127.689	13	Hex_{12} [3-AQ/M + K]$^+$
2289.726	3	Hex_{13} [3-AQ/M + K]$^+$
2451.763	- 3	Hex_{14} [3-AQ/M + K]$^+$

* Oligosaccharides are derivatized with 3-aminoquinoline (3-AQ) and ionized with H^+, Na^+ or K^+.

Supplementary Table 5. List of the assigned oligosaccharides for digested gum tragacanth corresponding to the **Supplementary Fig.-4** (i.e. gum tragacanth mass fingerprint).

Experimental mass [Da]	Δm [ppm]	Possible oligosaccharide*
843.225	- 3	Hex_2HexA_2 $[3AQ/M+Na]^+$
859.204	3	Hex_2HexA_2 $[3AQ/M+K]^+$
895.318	- 1	$Pent_2Hex_3$ $[3AQ/M+H]^+$
955.342	2	Hex_5 $[3AQ/M+H]^+$
957.296	1	$dHex_3HexA_2$ $[3AQ/M+Na]^+$
979.374	- 2	$Pent_3dHex_3$ $[3AQ/M+H]^+$
999.332	- 2	$Pent_2HexHexNAc_2$ $[3AQ/M+Na]^+$
1015.376	2	$Pent_2dHex_4$ $[3AQ/M+Na]^+$
1087.384	2	$PentHex_5$ $[3AQ/M+H]^+$
1103.357	4	$dHex_4HexA_2$ $[3AQ/M+Na]^+$
1119.329	2	$dHex_4HexA_2$ $[3AQ/M+K]^+$
1161.431	- 1	$Pent_2dHex_5$ $[3AQ/M+Na]^+$
1177.405	- 1	$Pent_2dHex_5$ $[3AQ/M+K]^+$

* Oligosaccharides are derivatized with 3-aminoquinoline (3-AQ) and ionized with H^+, Na^+ or K^+.

Supplementary Table 6. List of the fragment ions resulting from the MS/MS experiment of the [3-AQ/M + H]$^+$ ions at *m/z* 1055.360 and 1187.409 from the digested arabic gum (corresponding to spectra in **Fig. 2A** and **Fig. 2B** respectively).

Experimental mass [Da]	Δm [ppm]	Experimental mass [Da]	Δm [ppm]	Fragment ions*
Precursor ion *m/z* 1055.360		Precursor ion *m/z* 1187.409		
145.09	68	145.09	68	Y_0 (3-AQ) [M+H]$^+$**
277.11	36	277.10	72	Y_1 (Pent) [3-AQ/M+H]$^+$
307.14	32	307.13	-	Y_1’ (Hex) [3-AQ/M+H]$^+$
439.19	45	439.15	45	Y_2 (PentHex) [3-AQ/M+H]$^+$
469.20	42	469.21	63	Y_2' (Hex$_2$) [3-AQ/M+H]$^+$
601.24	33	601.22	-	Y_3 (PentHex$_2$) [3-AQ/M+H]$^+$
645.24	46	645.24	46	Y_3' (Hex$_2$HexA) [3-AQ/M+H]$^+$
733.28	13	733.30	40	Y_4 (Pent$_2$Hex$_2$) [3-AQ/M+H]$^+$
-	-	763.31	39	Y_4''' (PentHex$_3$) [3-AQ/M+H]$^+$
777.28	39	-	-	Y_4' (PentHex$_2$HexA) [3-AQ/M+H]$^+$
791.28	12	791.30	38	Y_4'' (Hex$_2$HexAdHex) [3-AQ/M+H]$^+$
-	-	865.32	12	Y_5''(Pent$_3$Hex$_2$)
909.31	11	909.34	44	Y_5 (Pent$_2$Hex$_2$HexA) [3-AQ/M+H]$^+$
-	-	909.34	11	Y_5 (PentHex$_3$dHex) [3-AQ/M+H]$^+$
923.32	11	923.32	11	Y_5' (PentHex$_2$ HexAdHex) [3-AQ/M+H]$^+$
-	-	1041.37	29	Y_6 (Pent$_3$Hex$_2$HexA) [3-AQ/M+H]$^+$
-	-	1041.37	9	Y_6 (Pent$_2$Hex$_3$dHex) [3-AQ/M+H]$^+$
-	-	1055.40	38	Y_6' (Pent$_2$Hex$_2$ HexAdHex) [3-AQ/M+H]$^+$
1055.360	0	-	-	Pent$_2$Hex$_2$dHexHexA [3-AQ/M+H]$^{+***}$
-	-	1187.409	8	Pent$_3$Hex$_2$dHexHexA [3-AQ/M+H]$^{+***}$
			- 17	Pent$_2$Hex$_3$dHex$_2$ [3-AQ/M+H]$^{+***}$

* Fragment ions are derivatized with 3-aminoquinoline (3-AQ) and ionized with H$^+$.
** The ion at m/z 145.11 (Y_0) corresponds to the protonated 3-aminoquinoline [M + H]$^+$.
*** Precursors ions at *m/z* 1055.360 and *m/z* 1187.409

Supplementary Table 7. List of the main oligosaccharides of the digested watercolor sample dating from 1870, Colour Box Charles Roberson & Co (The Metropolitan Museum of Art), corresponding to the **Fig. 3** (i.e. gum arabic mass fingerprint identified in the historic watercolor).

Experimental mass [Da]*	Δm [ppm]	Possible oligosaccharide*
629.216	- 6	HexdHexHexA $[3\text{-AQ/M+H}]^+$
667.184	6	HexdHexHexA $[3\text{-AQ/M+K}]^+$
733.260	- 13	$Pent_2Hex_2$ $[3\text{-AQ/M+H}]^+$
755.249	- 2	$Pent_2Hex_2$ $[3\text{-AQ/M+Na}]^+$
763.278	- 2	$PentHex_3$ $[3\text{-AQ/M+H}]^+$
771.218	- 3	$Pent_2Hex_2$ $[3\text{-AQ/M+K}]^+$
791.266	- 4	$Hex_2dHexHexA$ $[3\text{-AQ/M+H}]^+$
801.226	- 5	$PentHex_3$ $[3\text{-AQ/M+K}]^+$
813.245	- 6	$Hex_2dHexHexA$ $[3\text{-AQ/M+Na}]^+$
829.234	5	$Hex_2dHexHexA$ $[3\text{-AQ/M+K}]^+$
895.319	- 1	$Pent_2Hex_3$ $[3\text{-AQ/M+H}]^+$
917.298	- 2	$Pent_2Hex_3$ $[3\text{-AQ/M+Na}]^+$
923.307	- 3	$PentHex_2dHexHexA$ $[3\text{-AQ/M+H}]^+$
933.287	7	$Pent_2Hex_3$ $[3\text{-AQ/M+K}]^+$
945.296	- 5	$PentHex_2dHexHexA$ $[3\text{-AQ/M+Na}]^+$
961.275	5	$PentHex_2dHexHexA$ $[3\text{-AQ/M+K}]^+$
1055.367	7	$Pent_2Hex_2dHexHexA$ $[3\text{-AQ/M+H}]^+$
1077.335	- 4	$Pent_2Hex_2dHexHexA$ $[3\text{-AQ/M+Na}]^+$
1085.375	4	$PentHex_3dHexHexA$ $[3\text{-AQ/M+H}]^+$
1093.304	- 5	$Pent_2Hex_2dHexHexA$ $[3\text{-AQ/M+K}]^+$
1107.353	3	$PentHex_3dHexHexA$ $[3\text{-AQ/M+Na}]^+$
1123.322	1	$PentHex_3dHexHexA$ $[3\text{-AQ/M+K}]^+$
1187.406	5	$Pent_3Hex_2dHexHexA$ $[3\text{-AQ/M+H}]^+$
1187.406	- 20	$Pent_2Hex_3dHex_2$ $[3\text{-AQ/M+H}]^+$
1209.394	12	$Pent_3Hex_2dHexHexA$ $[3\text{-AQ/M+Na}]^+$
1209.394	- 21	$Pent_2Hex_3dHex_2$ $[3\text{-AQ/M+Na}]^+$
1217.413	3	$Pent_2Hex_3dHexHexA$ $[3\text{-AQ/M+H}]^+$
1225.363	10	$Pent_3Hex_2dHexHexA$ $[3\text{-AQ/M+K}]^+$
1225.363	- 22	$Pent_2Hex_3dHex_2$ $[3\text{-AQ/M+K}]^+$
1239.381	- 7	$Pent_2Hex_3dHexHexA$ $[3\text{-AQ/M+Na}]^+$
1255.370	0	$Pent_2Hex_3dHexHexA$ $[3\text{-AQ/M+K}]^+$

* Oligosaccharides are derivatized with 3-aminoquinoline (3-AQ) and ionized with H^+. Na^+ or K^+. The order of monosaccharides for each oligosaccharide is arbitrary and does not refer to its structure.
